# Supplementary figures and images for: PARG-deficient tumor cells have an increased dependence on EXO1/FEN1-mediated DNA repair
Source: EMBO J. 2024 Feb 15;43(6):6. doi: 10.1038/s44318-024-00043-2 (PMC10943112; doi:10.1038/s44318-024-00043-2)

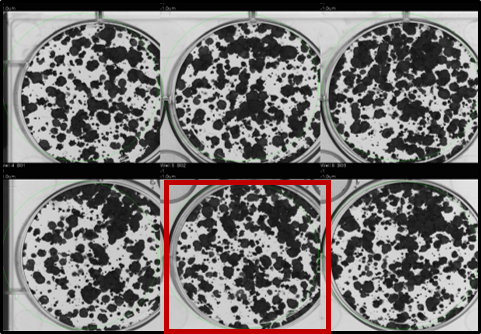

Supplement: Supplementary file 2 — Source Data Fig. 2 [file 44318_2024_43_MOESM2_ESM.zip › Figure 2/2C/Microscopic Images/KB2P NT-siExo1.tif]

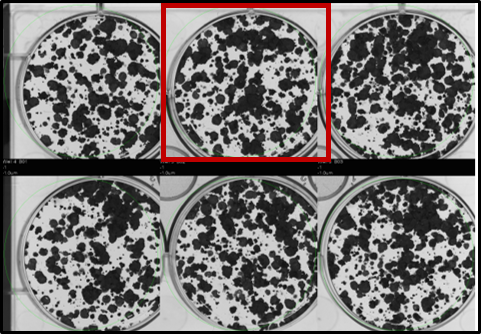

Supplement: Supplementary file 2 — Source Data Fig. 2 [file 44318_2024_43_MOESM2_ESM.zip › Figure 2/2C/Microscopic Images/KB2P NT-siNT.tif]

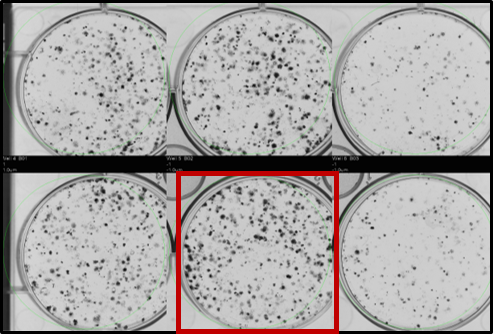

Supplement: Supplementary file 2 — Source Data Fig. 2 [file 44318_2024_43_MOESM2_ESM.zip › Figure 2/2C/Microscopic Images/KB2P-P2 siExo1.tif]

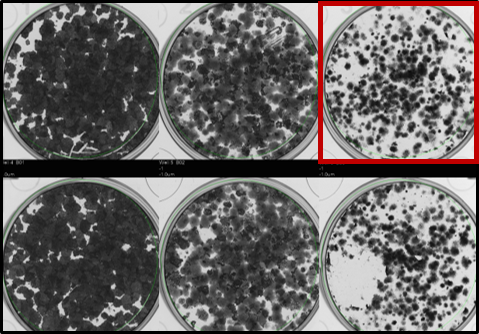

Supplement: Supplementary file 2 — Source Data Fig. 2 [file 44318_2024_43_MOESM2_ESM.zip › Figure 2/2C/Microscopic Images/KB2P-P2 siNT.tif]

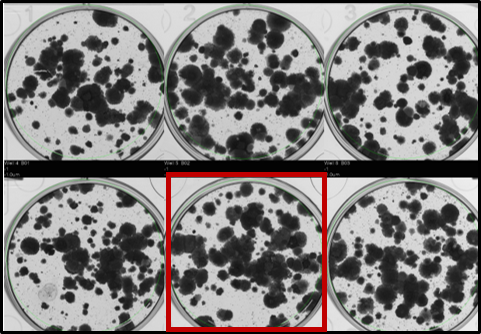

Supplement: Supplementary file 2 — Source Data Fig. 2 [file 44318_2024_43_MOESM2_ESM.zip › Figure 2/2C/Microscopic Images/KP NT-siExo1.tif]

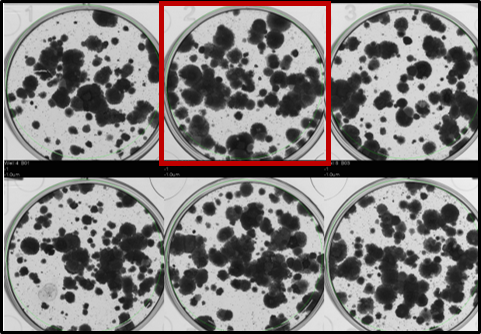

Supplement: Supplementary file 2 — Source Data Fig. 2 [file 44318_2024_43_MOESM2_ESM.zip › Figure 2/2C/Microscopic Images/KP NT-siNT.tif]

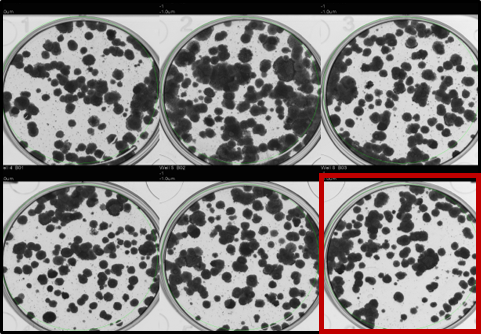

Supplement: Supplementary file 2 — Source Data Fig. 2 [file 44318_2024_43_MOESM2_ESM.zip › Figure 2/2C/Microscopic Images/KP-P1-siExo1.tif]

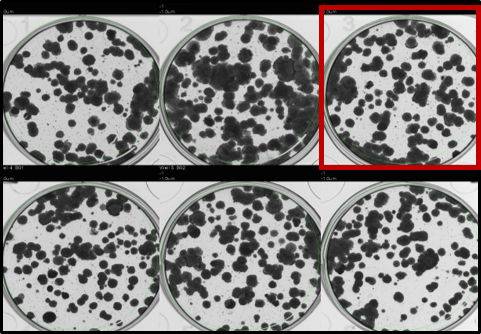

Supplement: Supplementary file 2 — Source Data Fig. 2 [file 44318_2024_43_MOESM2_ESM.zip › Figure 2/2C/Microscopic Images/KP-P1-siNT.tif]

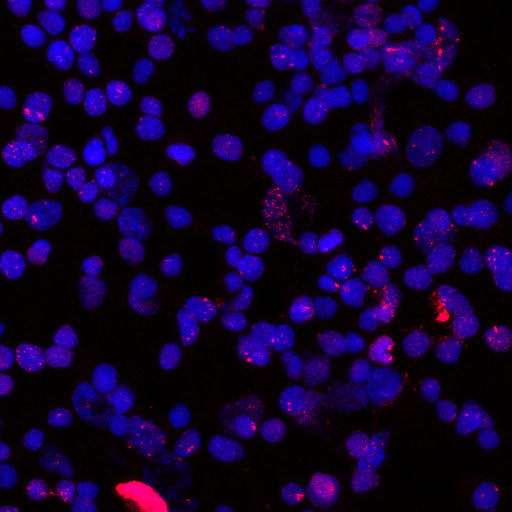

Supplement: Supplementary file 3 — Source Data Fig. 3 [file 44318_2024_43_MOESM3_ESM.zip › Figure 3/3A/Microscopic Images/KB2P-NT siExo1.tif]

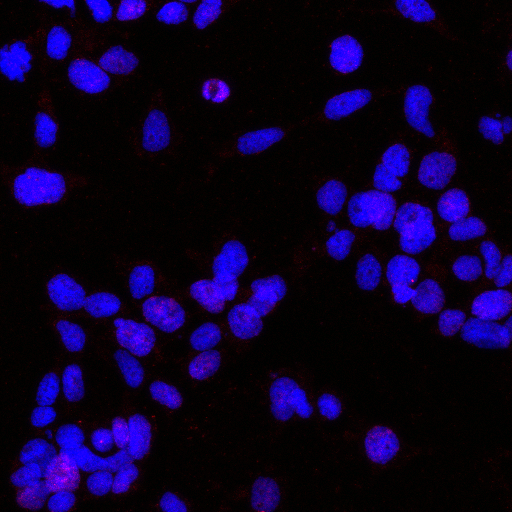

Supplement: Supplementary file 3 — Source Data Fig. 3 [file 44318_2024_43_MOESM3_ESM.zip › Figure 3/3A/Microscopic Images/KB2P-NT siNT.tif]

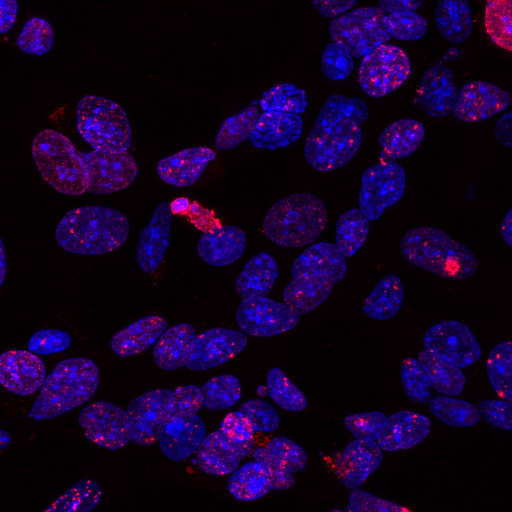

Supplement: Supplementary file 3 — Source Data Fig. 3 [file 44318_2024_43_MOESM3_ESM.zip › Figure 3/3A/Microscopic Images/KB2P-P2 siExo1.tif]

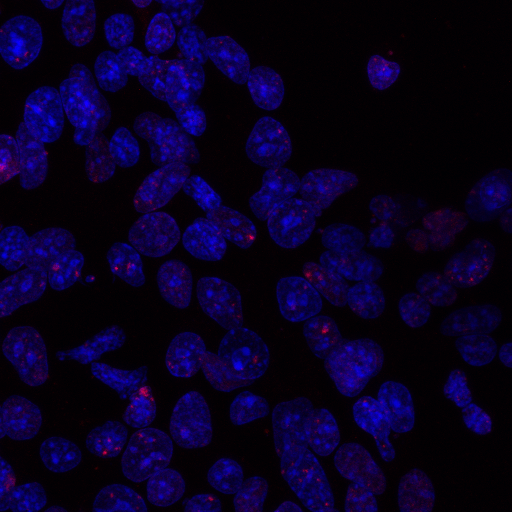

Supplement: Supplementary file 3 — Source Data Fig. 3 [file 44318_2024_43_MOESM3_ESM.zip › Figure 3/3A/Microscopic Images/KB2P-P2 siNT.tif]

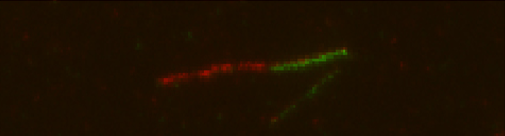

Supplement: Supplementary file 3 — Source Data Fig. 3 [file 44318_2024_43_MOESM3_ESM.zip › Figure 3/3E/Microscopic images/KB2P-NT siExo1.tif]

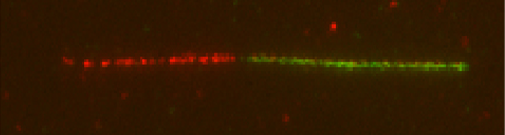

Supplement: Supplementary file 3 — Source Data Fig. 3 [file 44318_2024_43_MOESM3_ESM.zip › Figure 3/3E/Microscopic images/KB2P-NT siNT.tif]

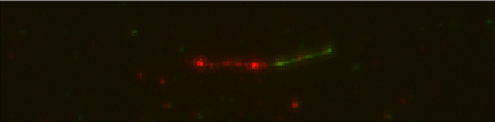

Supplement: Supplementary file 3 — Source Data Fig. 3 [file 44318_2024_43_MOESM3_ESM.zip › Figure 3/3E/Microscopic images/KB2P-P2 siExo1.tif]

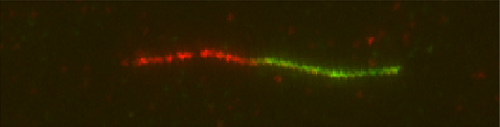

Supplement: Supplementary file 3 — Source Data Fig. 3 [file 44318_2024_43_MOESM3_ESM.zip › Figure 3/3E/Microscopic images/KB2P-P2 siNT.tif]

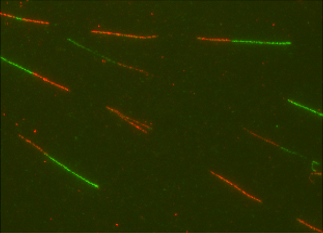

Supplement: Supplementary file 3 — Source Data Fig. 3 [file 44318_2024_43_MOESM3_ESM.zip › Figure 3/3F/Microscopic images/KB2P-NT siExo1.tif]

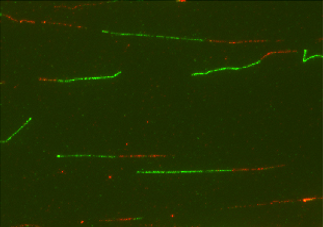

Supplement: Supplementary file 3 — Source Data Fig. 3 [file 44318_2024_43_MOESM3_ESM.zip › Figure 3/3F/Microscopic images/KB2P-NT siNT.tif]

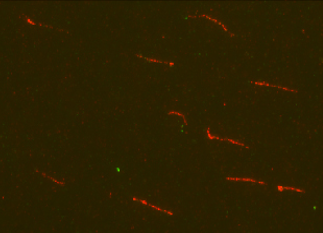

Supplement: Supplementary file 3 — Source Data Fig. 3 [file 44318_2024_43_MOESM3_ESM.zip › Figure 3/3F/Microscopic images/KB2P-P2 siExo1.tif]

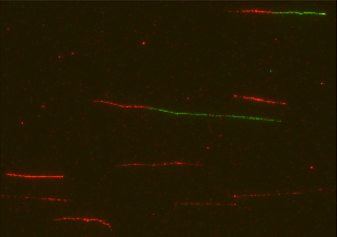

Supplement: Supplementary file 3 — Source Data Fig. 3 [file 44318_2024_43_MOESM3_ESM.zip › Figure 3/3F/Microscopic images/KB2P-P2 siNT.tif]

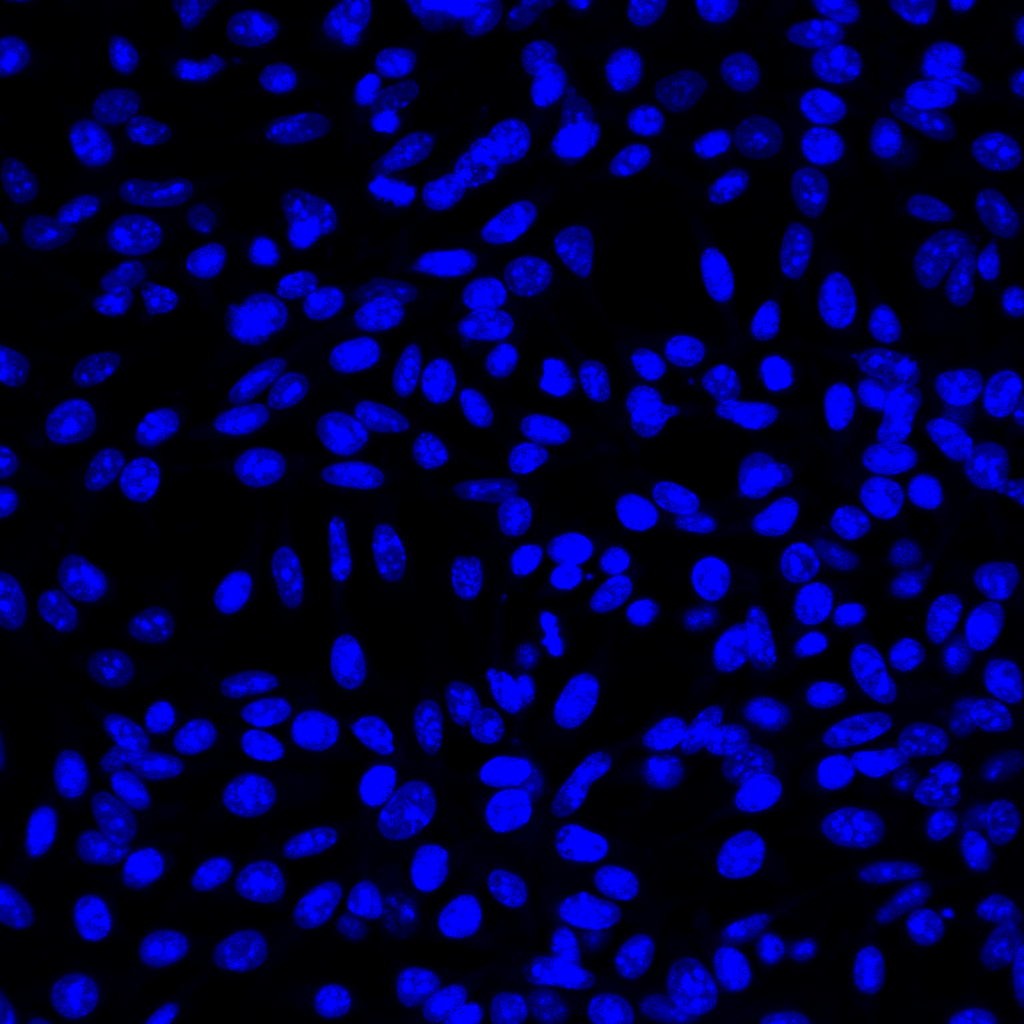

Supplement: Supplementary file 4 — Source Data Fig. 4 [file 44318_2024_43_MOESM4_ESM.zip › Figure 4/4C/MIcroscopic images/KB2P-NT DMSO DAPI.tif]

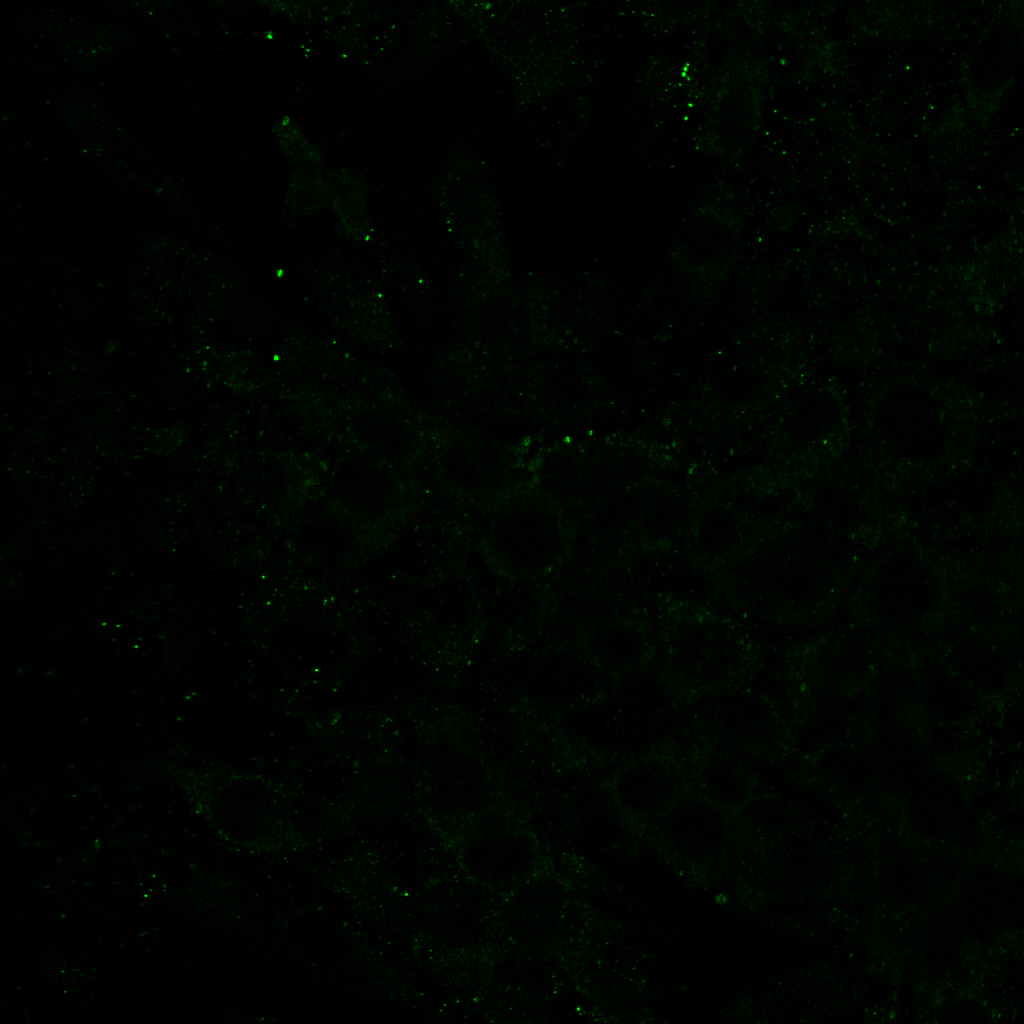

Supplement: Supplementary file 4 — Source Data Fig. 4 [file 44318_2024_43_MOESM4_ESM.zip › Figure 4/4C/MIcroscopic images/KB2P-NT DMSO PAR.tif]

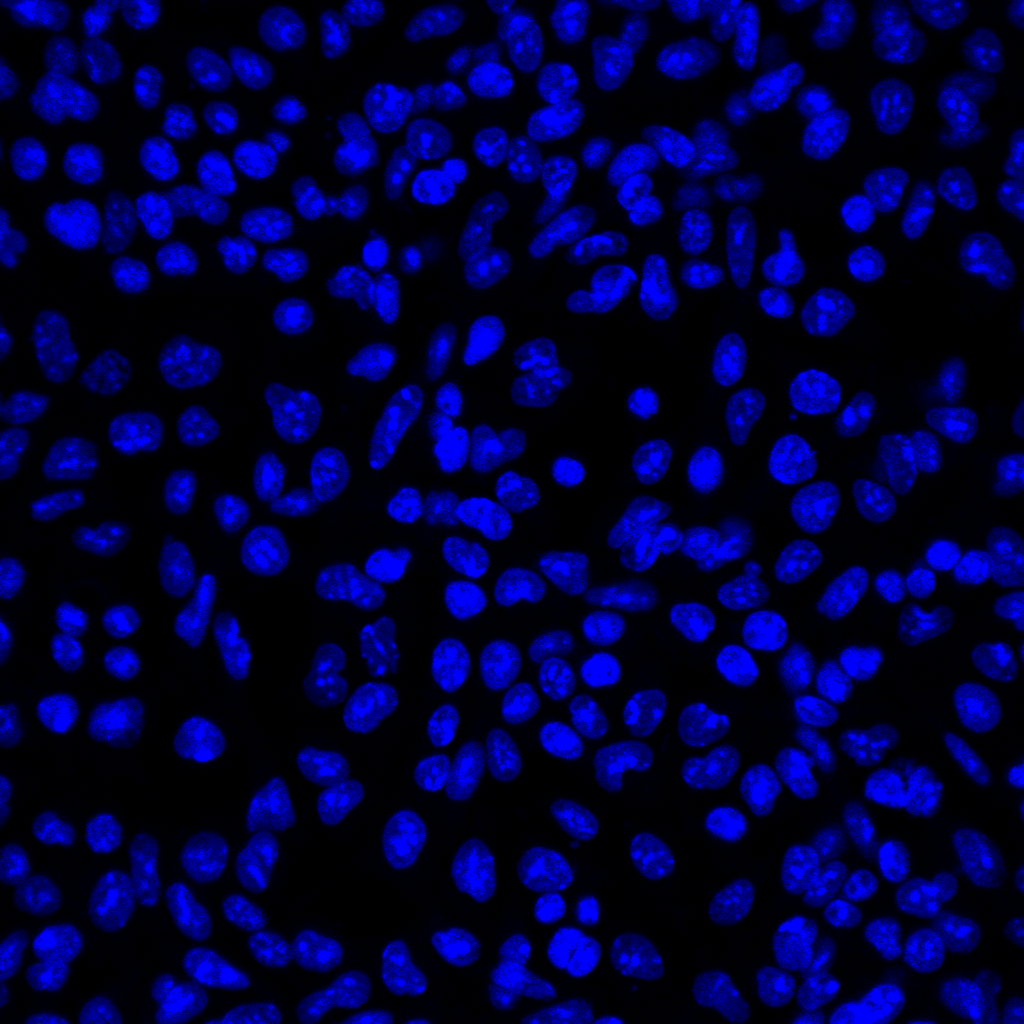

Supplement: Supplementary file 4 — Source Data Fig. 4 [file 44318_2024_43_MOESM4_ESM.zip › Figure 4/4C/MIcroscopic images/KB2P-NT EME DAPI.tif]

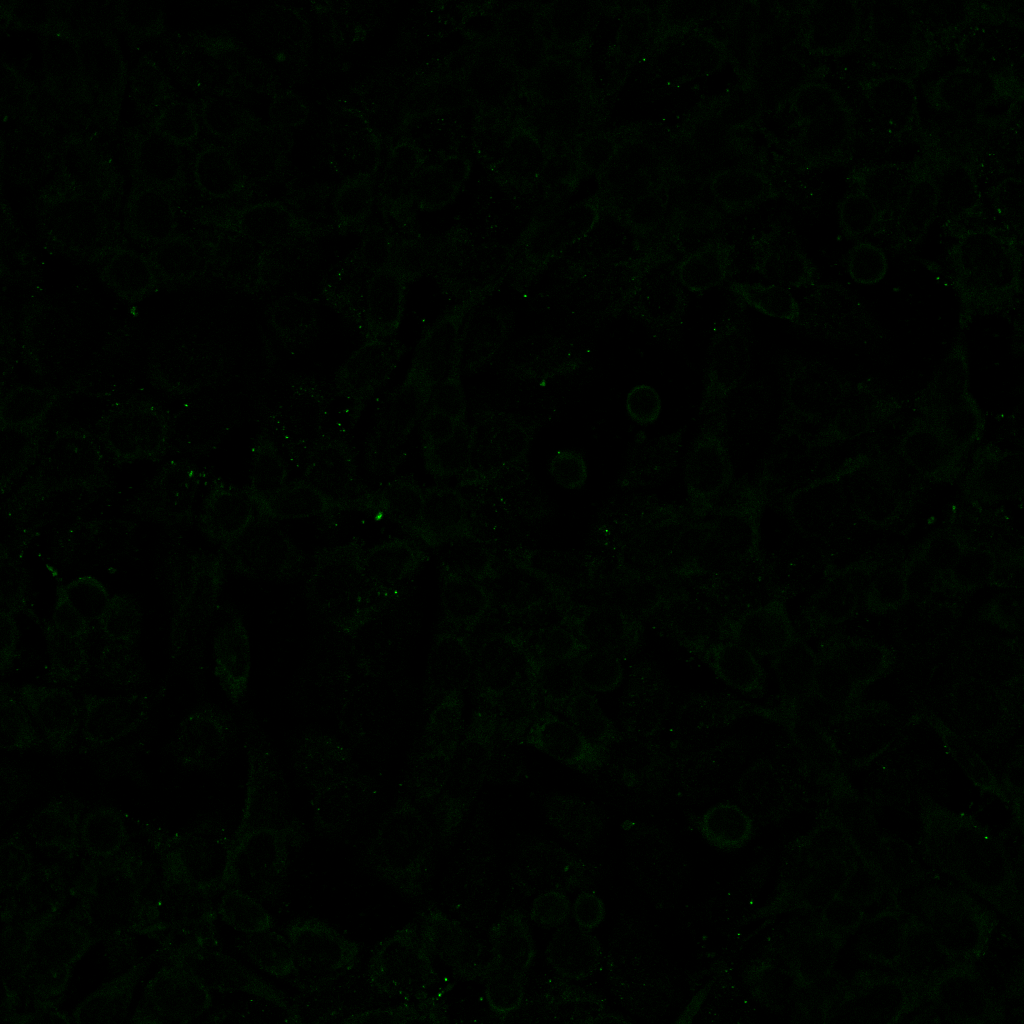

Supplement: Supplementary file 4 — Source Data Fig. 4 [file 44318_2024_43_MOESM4_ESM.zip › Figure 4/4C/MIcroscopic images/KB2P-NT EME PAR.tif]

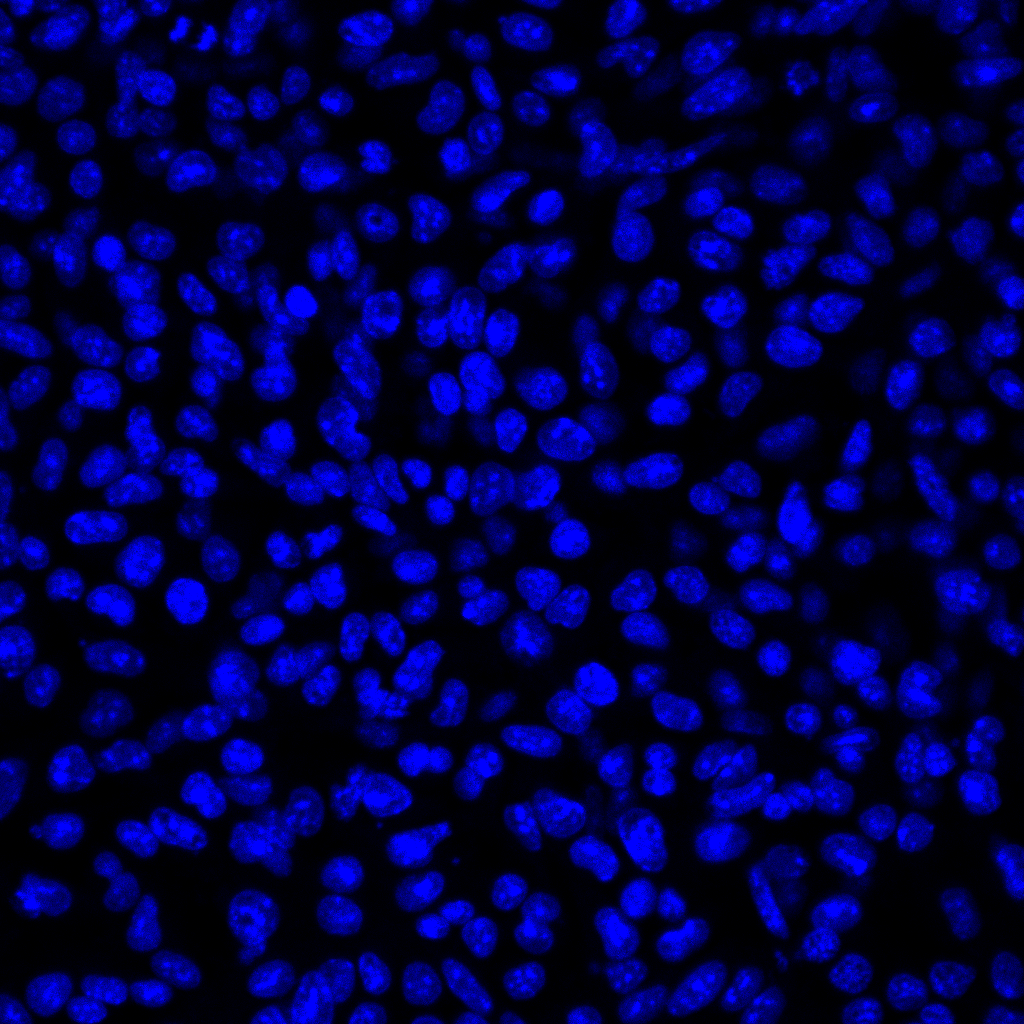

Supplement: Supplementary file 4 — Source Data Fig. 4 [file 44318_2024_43_MOESM4_ESM.zip › Figure 4/4C/MIcroscopic images/KB2P-NT LNT1 DAPI.tif]

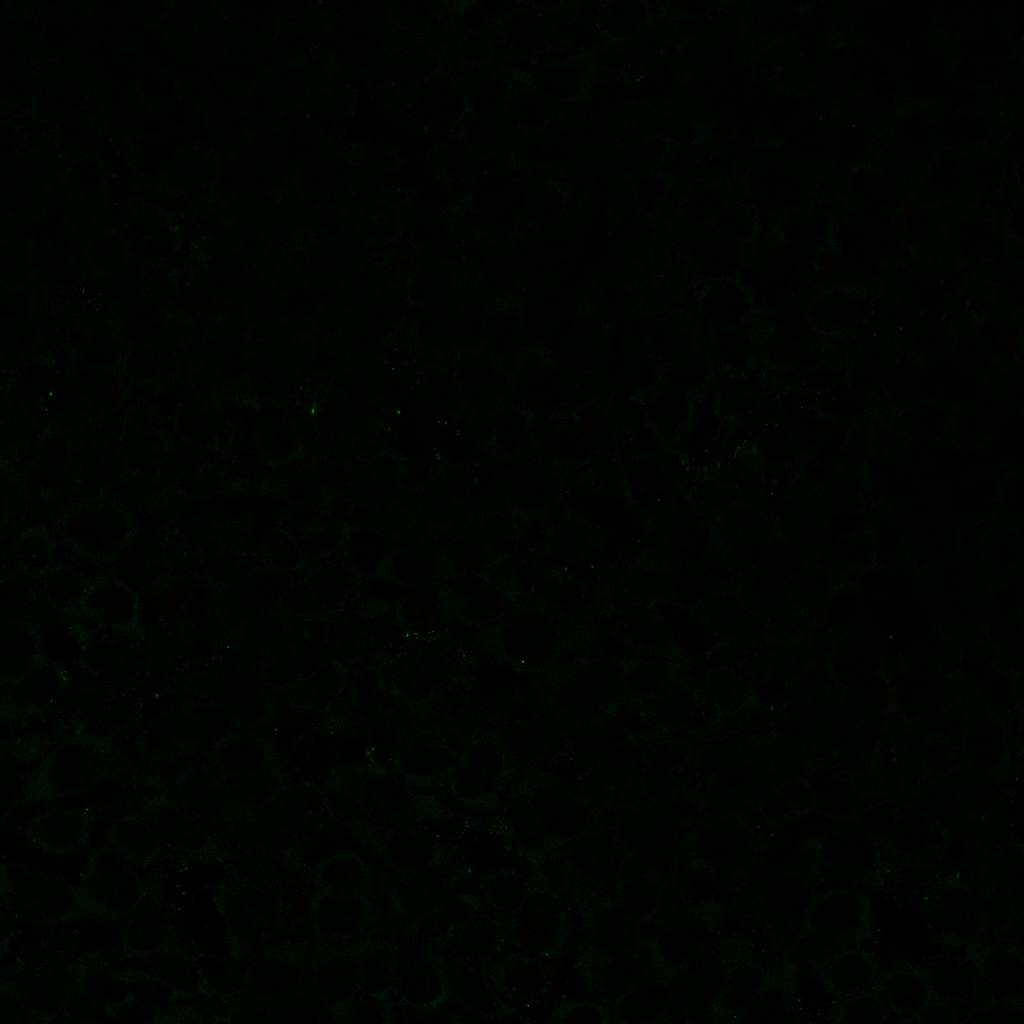

Supplement: Supplementary file 4 — Source Data Fig. 4 [file 44318_2024_43_MOESM4_ESM.zip › Figure 4/4C/MIcroscopic images/KB2P-NT LNT1 PAR.tif]

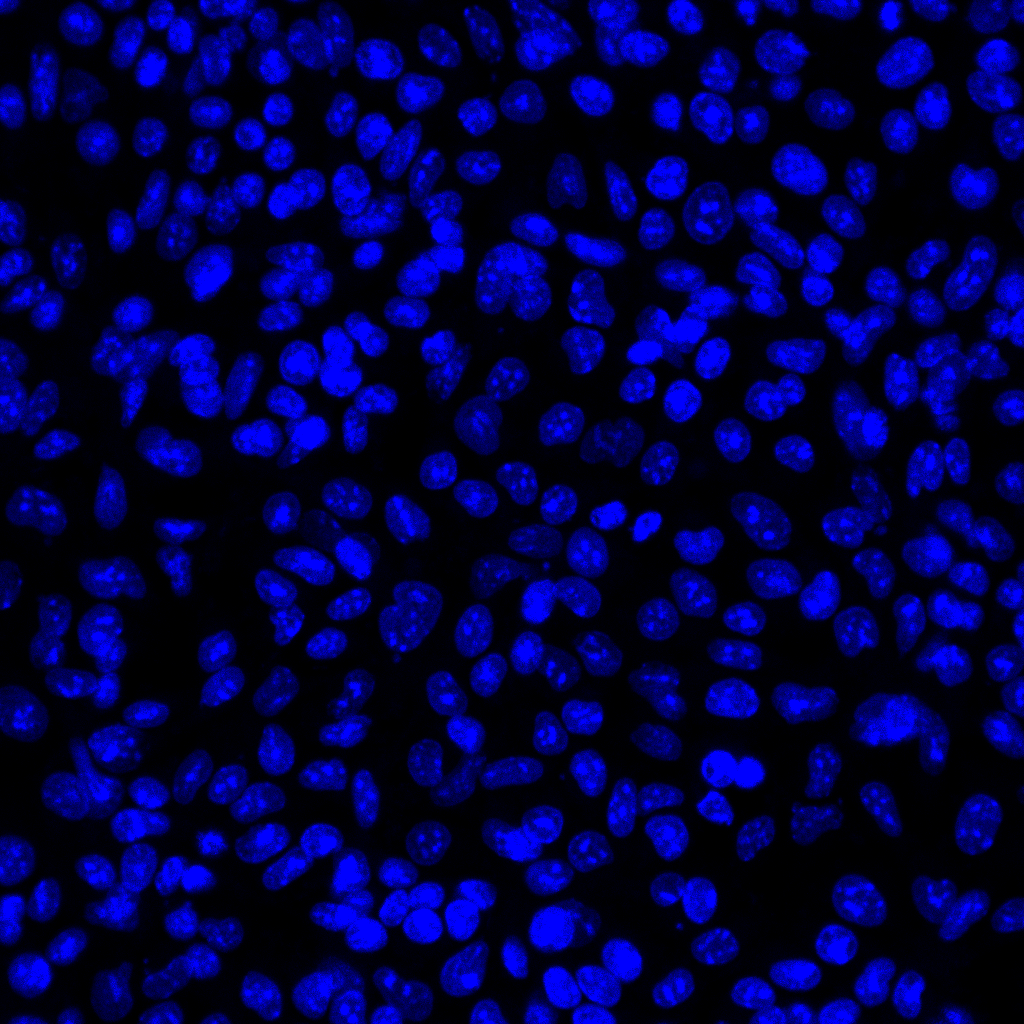

Supplement: Supplementary file 4 — Source Data Fig. 4 [file 44318_2024_43_MOESM4_ESM.zip › Figure 4/4C/MIcroscopic images/KB2P-NT LNT1+EME DAPI.tif]

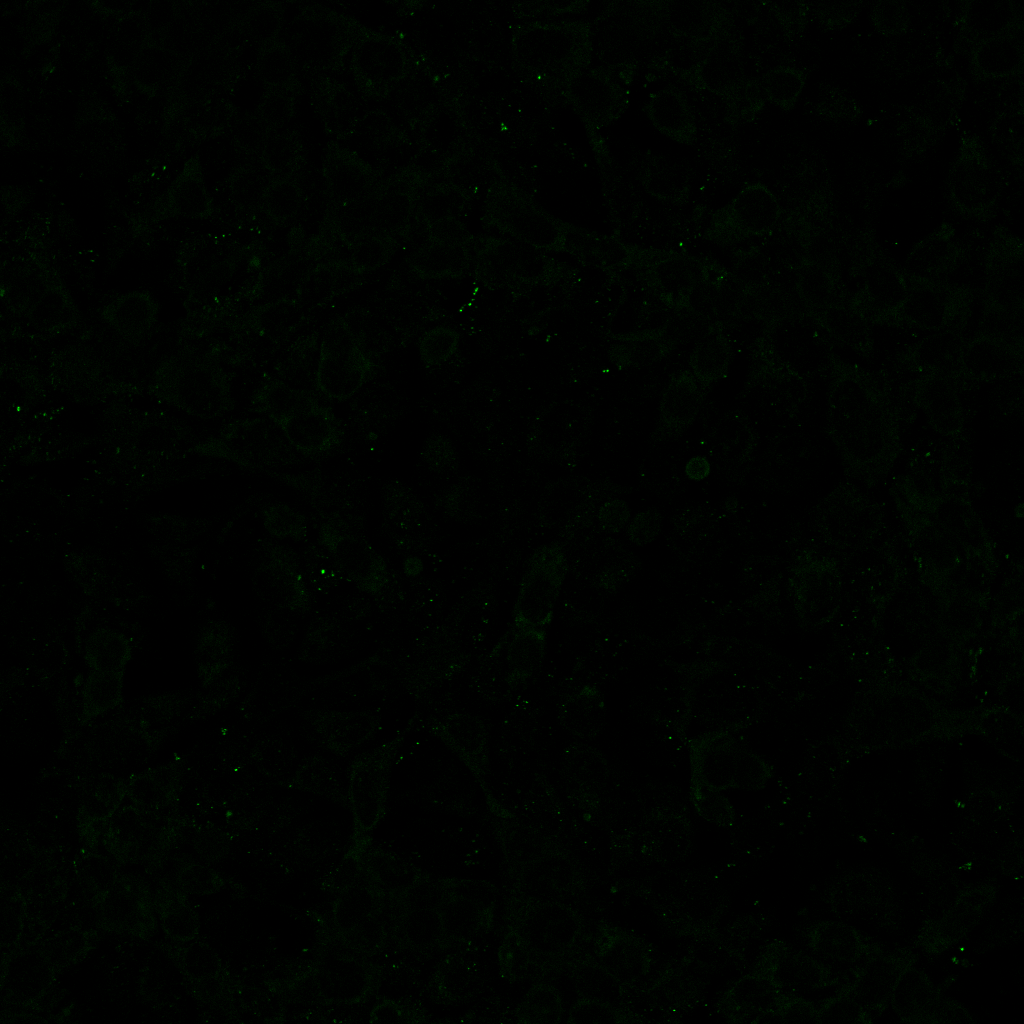

Supplement: Supplementary file 4 — Source Data Fig. 4 [file 44318_2024_43_MOESM4_ESM.zip › Figure 4/4C/MIcroscopic images/KB2P-NT LNT1+EME PAR.tif]

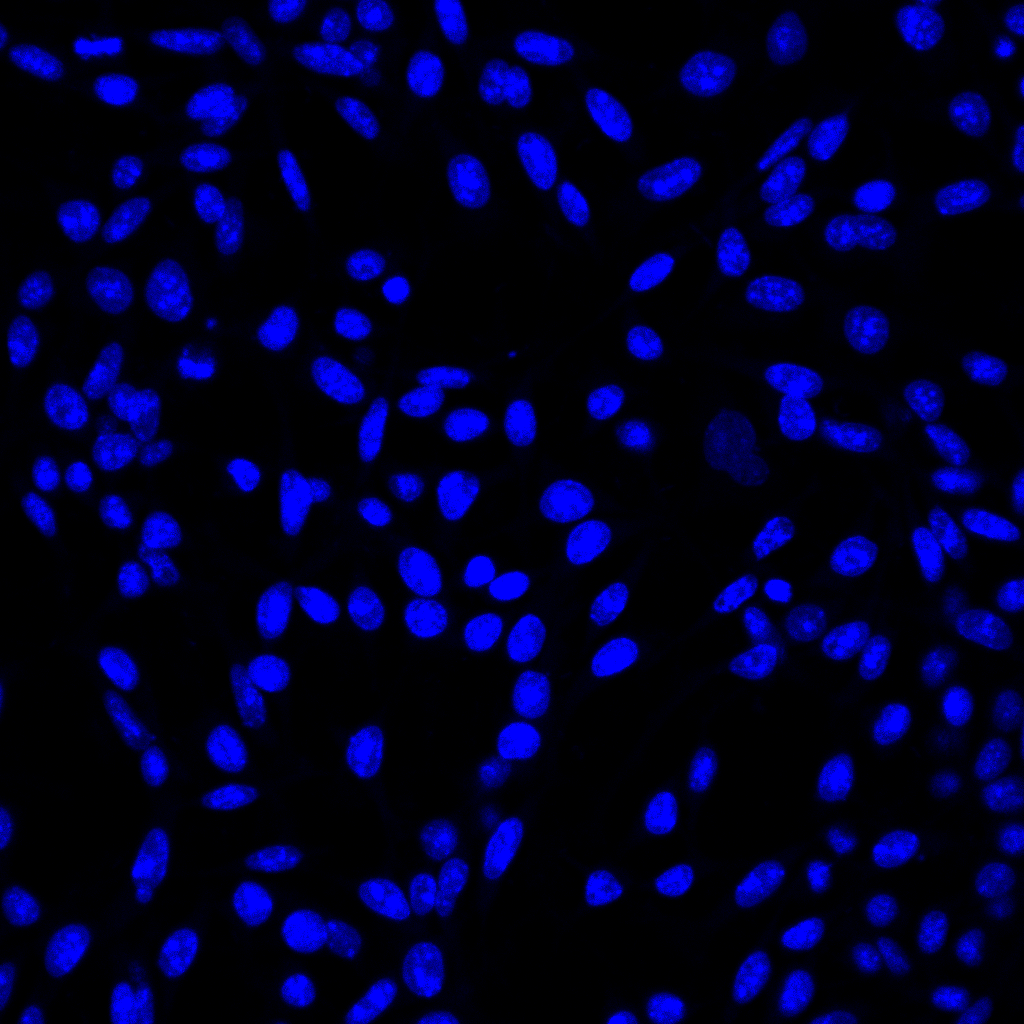

Supplement: Supplementary file 4 — Source Data Fig. 4 [file 44318_2024_43_MOESM4_ESM.zip › Figure 4/4C/MIcroscopic images/KB2P-P2 DMSO DAPI.tif]

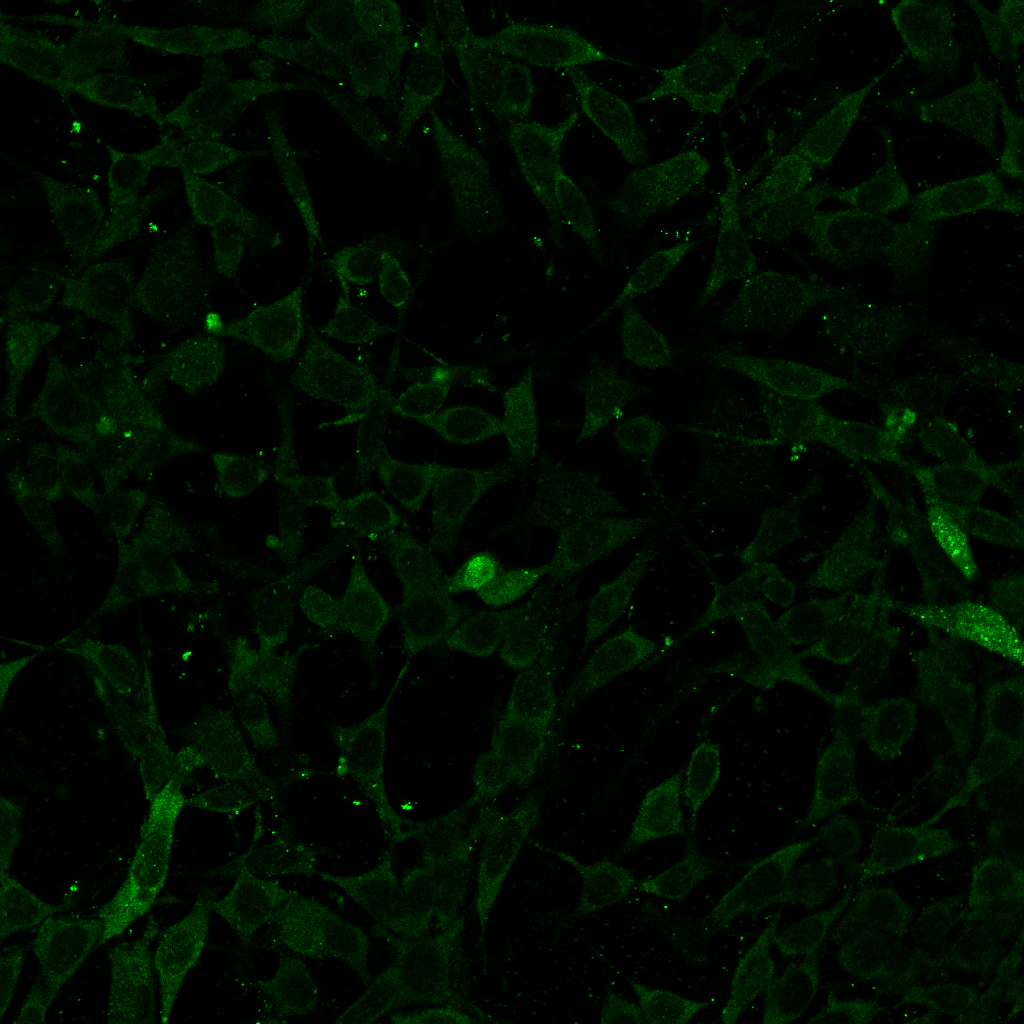

Supplement: Supplementary file 4 — Source Data Fig. 4 [file 44318_2024_43_MOESM4_ESM.zip › Figure 4/4C/MIcroscopic images/KB2P-P2 DMSO PAR.tif]

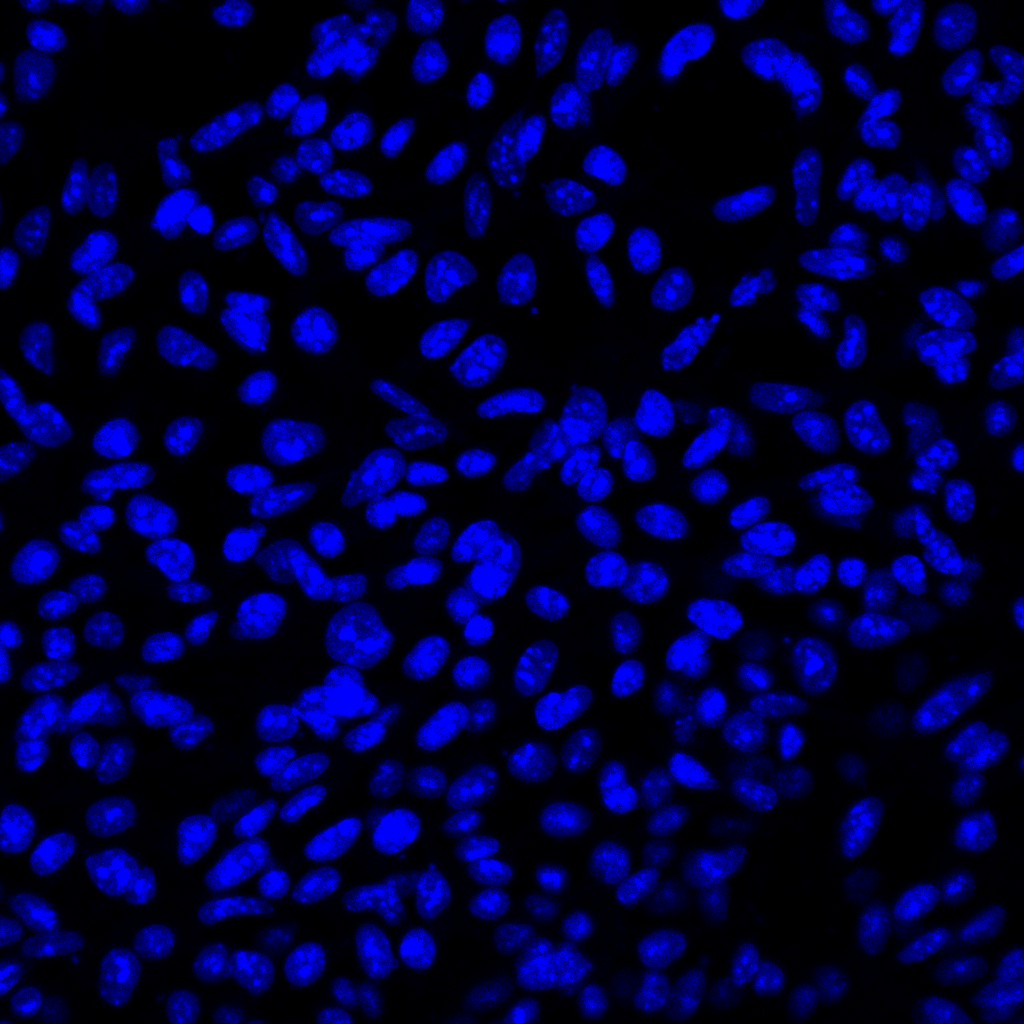

Supplement: Supplementary file 4 — Source Data Fig. 4 [file 44318_2024_43_MOESM4_ESM.zip › Figure 4/4C/MIcroscopic images/KB2P-P2 EME DAPI.tif]

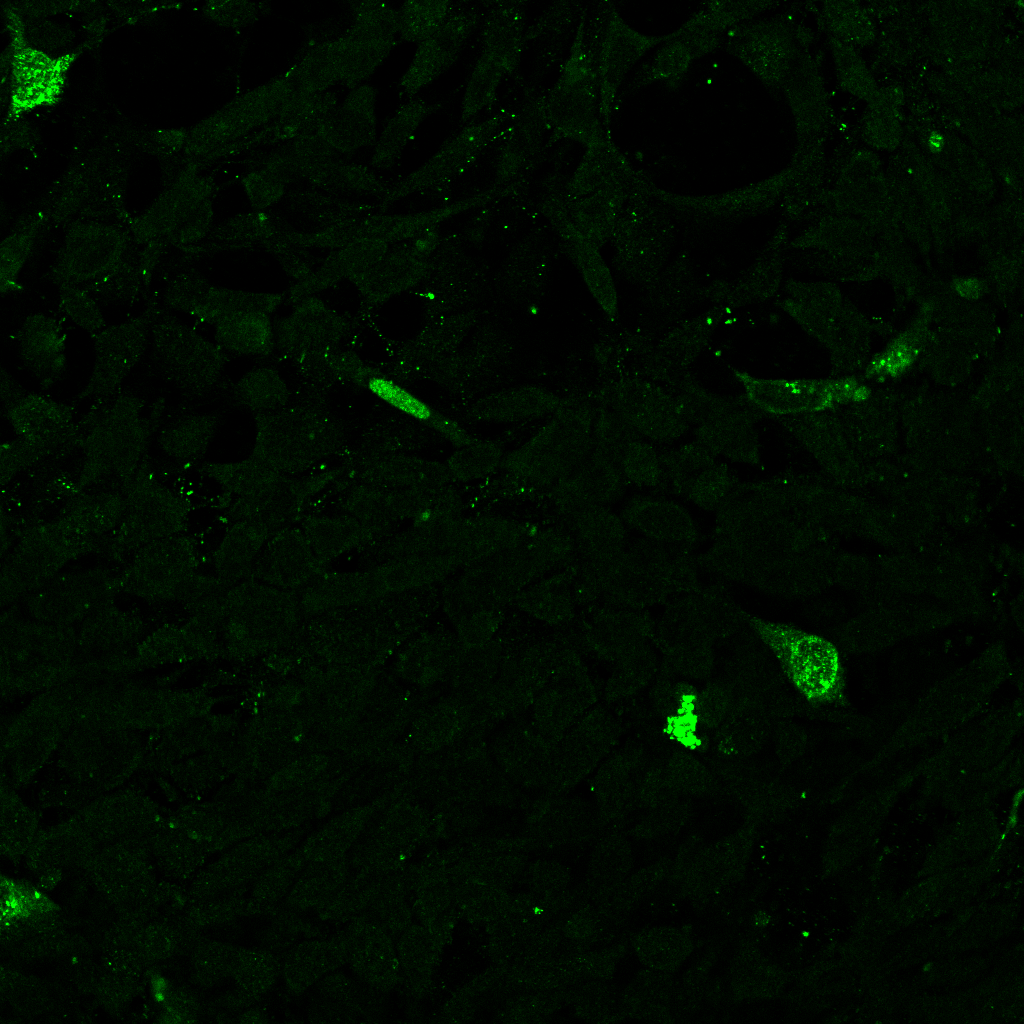

Supplement: Supplementary file 4 — Source Data Fig. 4 [file 44318_2024_43_MOESM4_ESM.zip › Figure 4/4C/MIcroscopic images/KB2P-P2 EME PAR.tif]

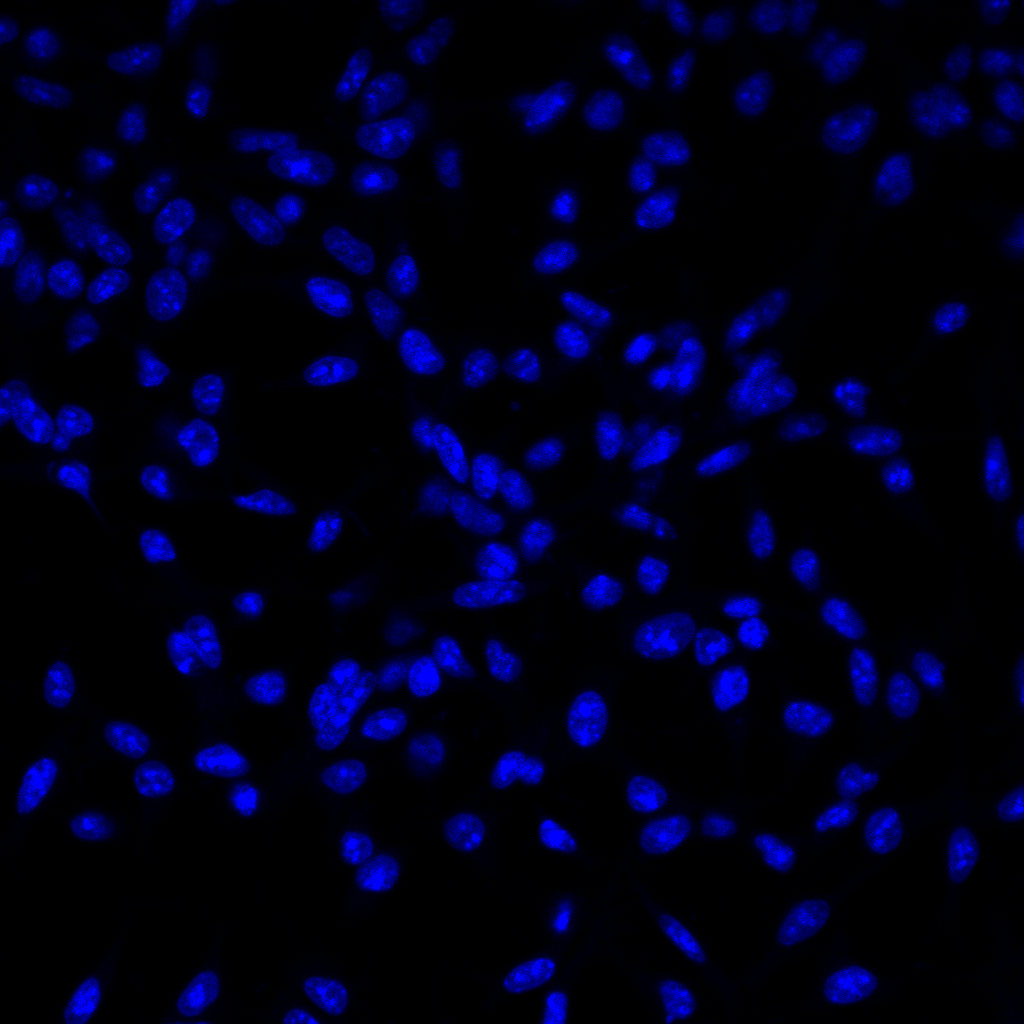

Supplement: Supplementary file 4 — Source Data Fig. 4 [file 44318_2024_43_MOESM4_ESM.zip › Figure 4/4C/MIcroscopic images/KB2P-P2 LNT1 DAPI.tif]

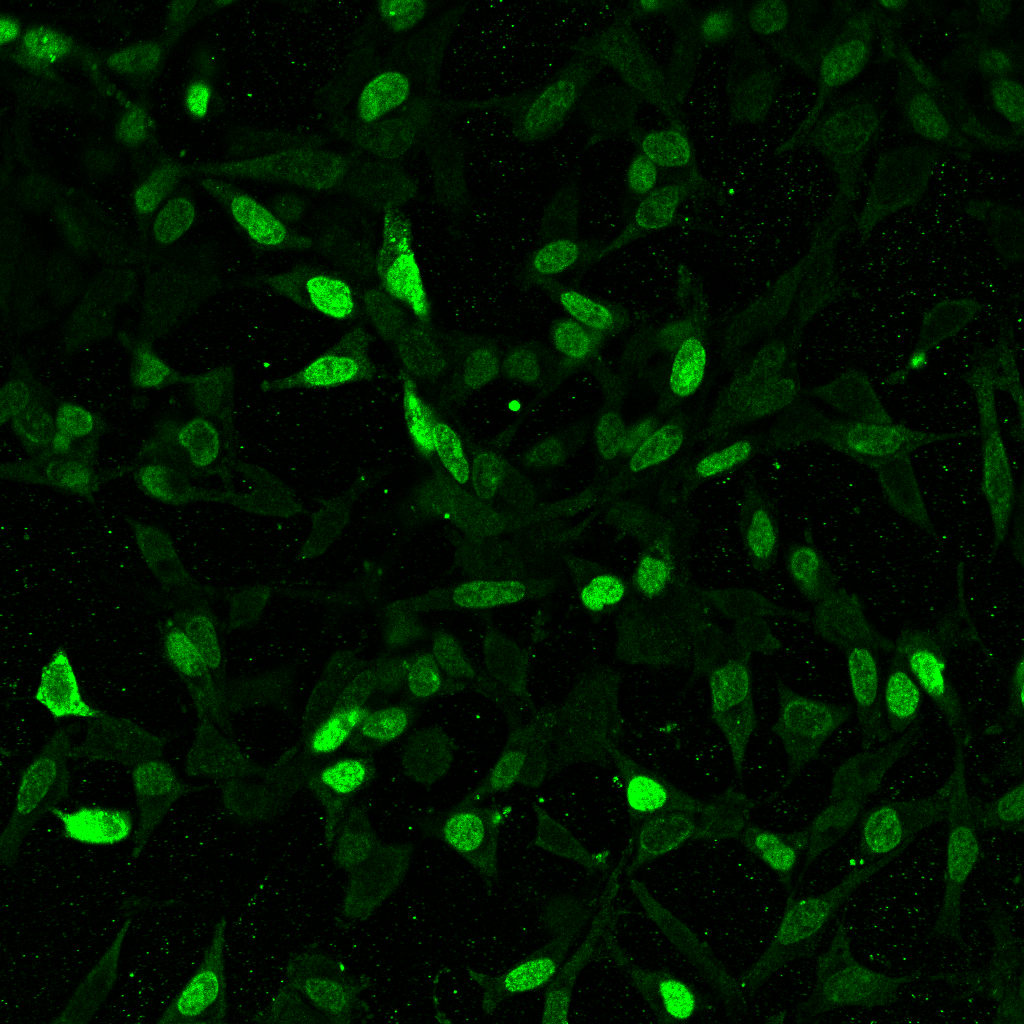

Supplement: Supplementary file 4 — Source Data Fig. 4 [file 44318_2024_43_MOESM4_ESM.zip › Figure 4/4C/MIcroscopic images/KB2P-P2 LNT1 PAR.tif]

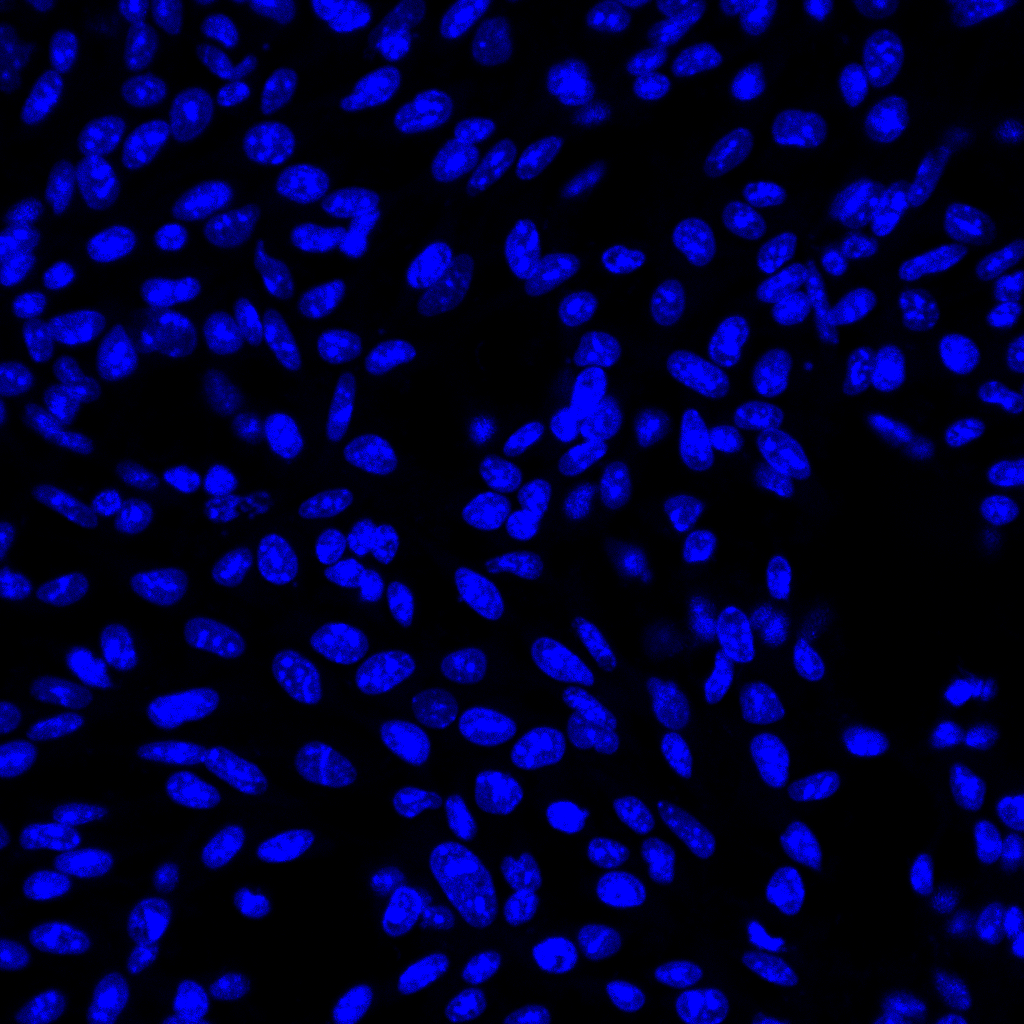

Supplement: Supplementary file 4 — Source Data Fig. 4 [file 44318_2024_43_MOESM4_ESM.zip › Figure 4/4C/MIcroscopic images/KB2P-P2 LNT1+EME DAPI DAPI.tif]

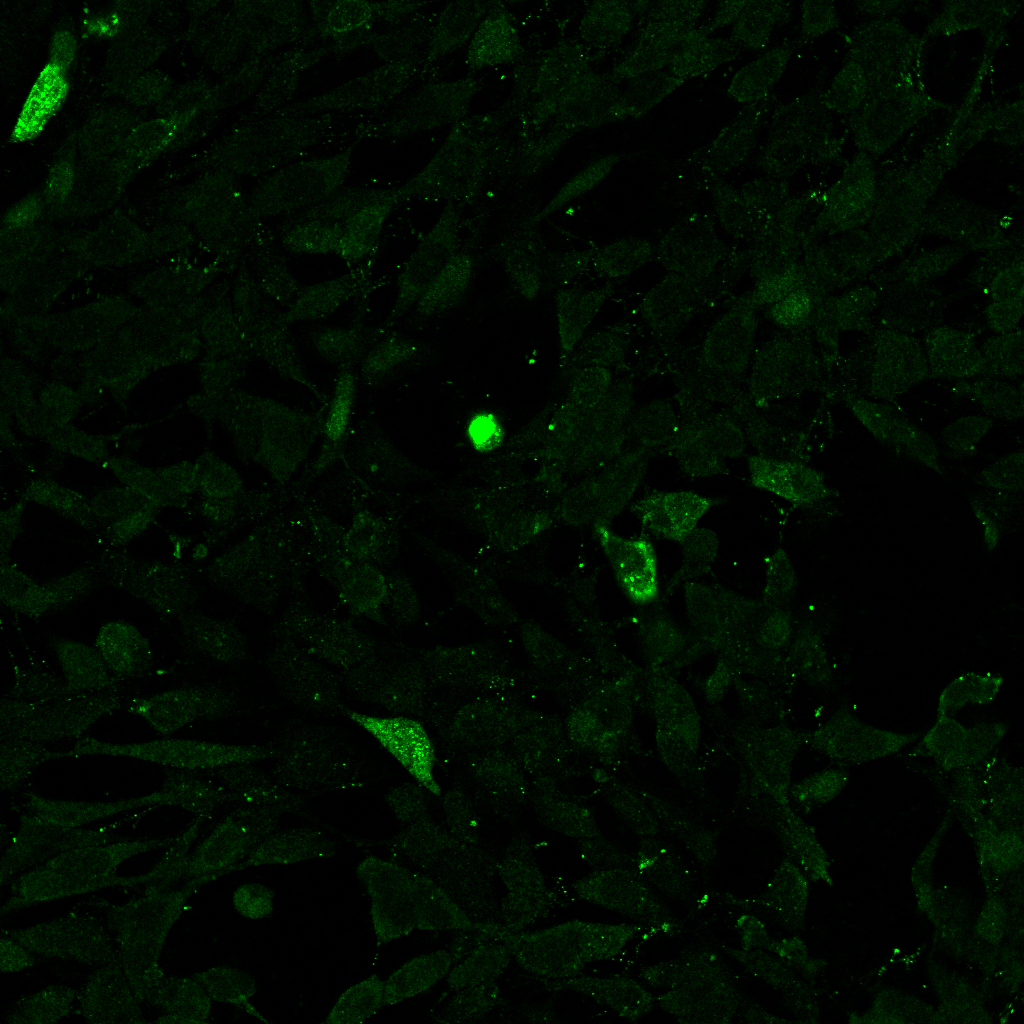

Supplement: Supplementary file 4 — Source Data Fig. 4 [file 44318_2024_43_MOESM4_ESM.zip › Figure 4/4C/MIcroscopic images/KB2P-P2 LNT1+EME PAR.tif]

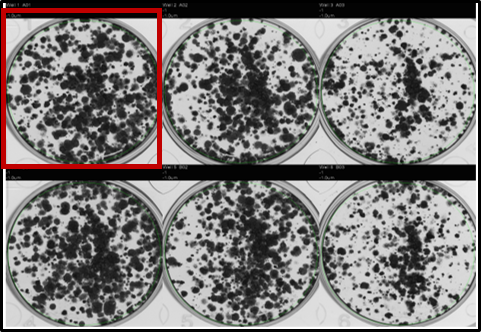

Supplement: Supplementary file 5 — Source Data Fig. 5 [file 44318_2024_43_MOESM5_ESM.zip › EMBOJ-2023-114851_SourceData_Figure5/Figure 5/5A/Images/KB2P NT 0nM.tif]

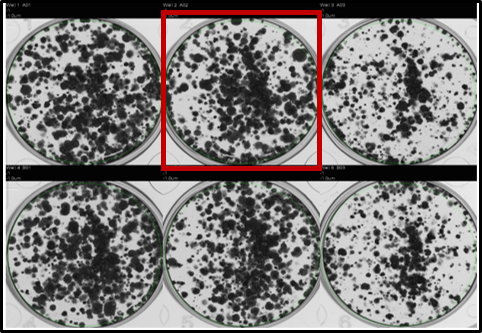

Supplement: Supplementary file 5 — Source Data Fig. 5 [file 44318_2024_43_MOESM5_ESM.zip › EMBOJ-2023-114851_SourceData_Figure5/Figure 5/5A/Images/KB2P NT 2.5nM.tif]

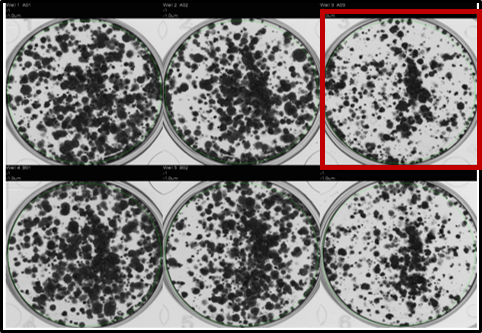

Supplement: Supplementary file 5 — Source Data Fig. 5 [file 44318_2024_43_MOESM5_ESM.zip › EMBOJ-2023-114851_SourceData_Figure5/Figure 5/5A/Images/KB2P NT 5nM.tif]

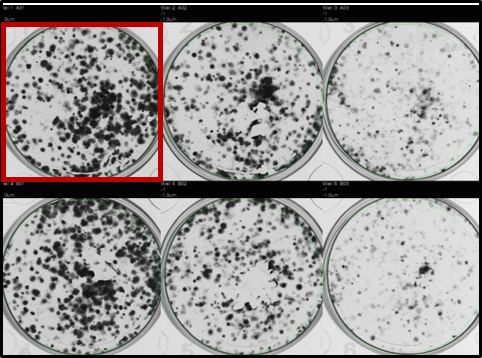

Supplement: Supplementary file 5 — Source Data Fig. 5 [file 44318_2024_43_MOESM5_ESM.zip › EMBOJ-2023-114851_SourceData_Figure5/Figure 5/5A/Images/KB2P-P2 0nM.tif]

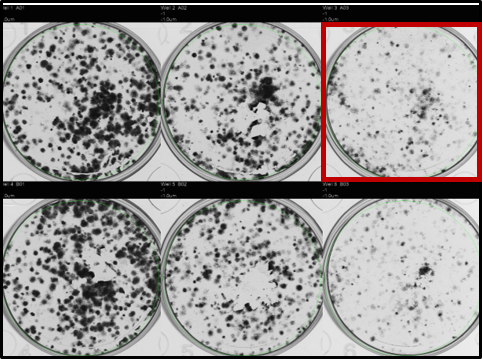

Supplement: Supplementary file 5 — Source Data Fig. 5 [file 44318_2024_43_MOESM5_ESM.zip › EMBOJ-2023-114851_SourceData_Figure5/Figure 5/5A/Images/KB2P-P2 2.5nM.tif]

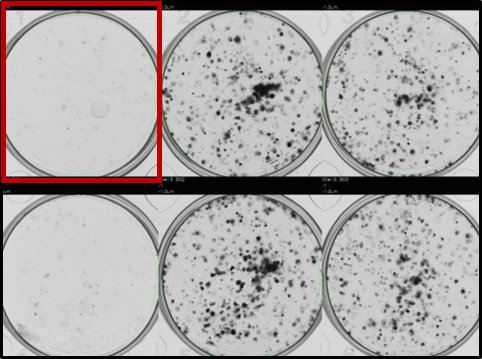

Supplement: Supplementary file 5 — Source Data Fig. 5 [file 44318_2024_43_MOESM5_ESM.zip › EMBOJ-2023-114851_SourceData_Figure5/Figure 5/5A/Images/KB2P-P2 5nM.tif]

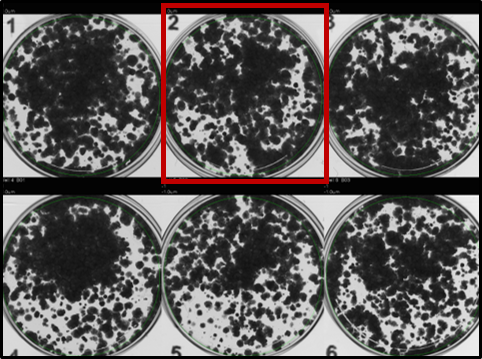

Supplement: Supplementary file 5 — Source Data Fig. 5 [file 44318_2024_43_MOESM5_ESM.zip › EMBOJ-2023-114851_SourceData_Figure5/Figure 5/5A/Images/KP NT 0nM.tif]

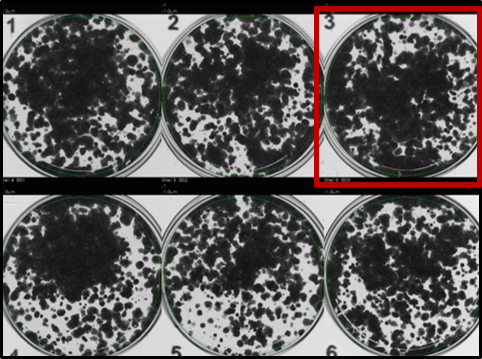

Supplement: Supplementary file 5 — Source Data Fig. 5 [file 44318_2024_43_MOESM5_ESM.zip › EMBOJ-2023-114851_SourceData_Figure5/Figure 5/5A/Images/KP NT 2.5nM.tif]

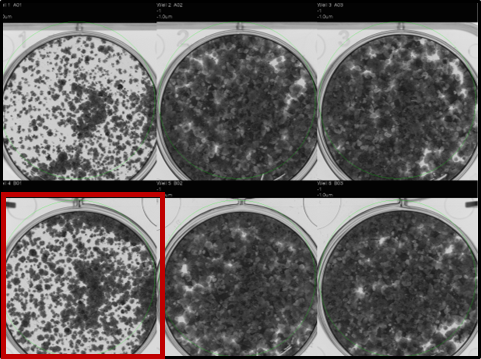

Supplement: Supplementary file 5 — Source Data Fig. 5 [file 44318_2024_43_MOESM5_ESM.zip › EMBOJ-2023-114851_SourceData_Figure5/Figure 5/5A/Images/KP NT 5nM.tif]

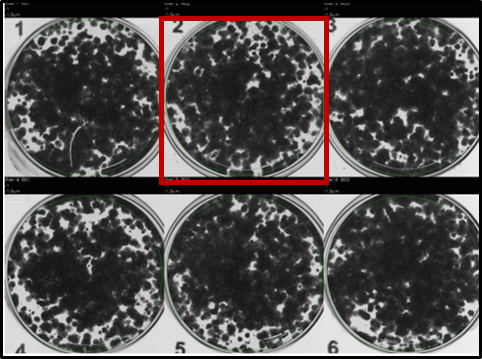

Supplement: Supplementary file 5 — Source Data Fig. 5 [file 44318_2024_43_MOESM5_ESM.zip › EMBOJ-2023-114851_SourceData_Figure5/Figure 5/5A/Images/KP-P1 0nM.tif]

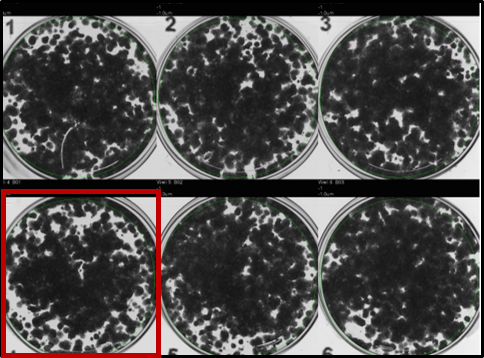

Supplement: Supplementary file 5 — Source Data Fig. 5 [file 44318_2024_43_MOESM5_ESM.zip › EMBOJ-2023-114851_SourceData_Figure5/Figure 5/5A/Images/KP-P1 2.5nM.tif]

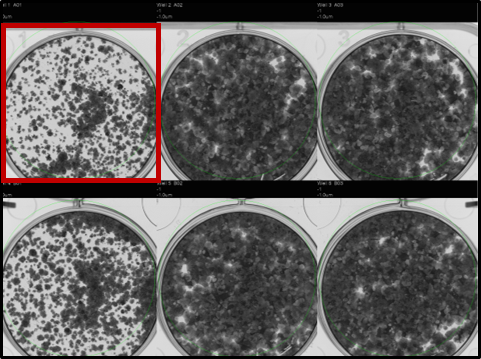

Supplement: Supplementary file 5 — Source Data Fig. 5 [file 44318_2024_43_MOESM5_ESM.zip › EMBOJ-2023-114851_SourceData_Figure5/Figure 5/5A/Images/KP-P1 5nM.tif]

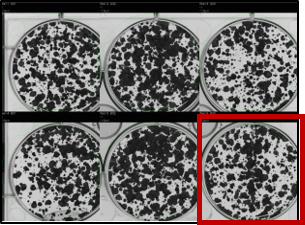

Supplement: Supplementary file 5 — Source Data Fig. 5 [file 44318_2024_43_MOESM5_ESM.zip › EMBOJ-2023-114851_SourceData_Figure5/Figure 5/5B/Images/KB2P-NT siFen1.tif]

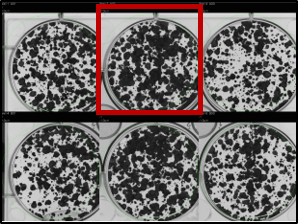

Supplement: Supplementary file 5 — Source Data Fig. 5 [file 44318_2024_43_MOESM5_ESM.zip › EMBOJ-2023-114851_SourceData_Figure5/Figure 5/5B/Images/KB2P-NT siNT.tif]

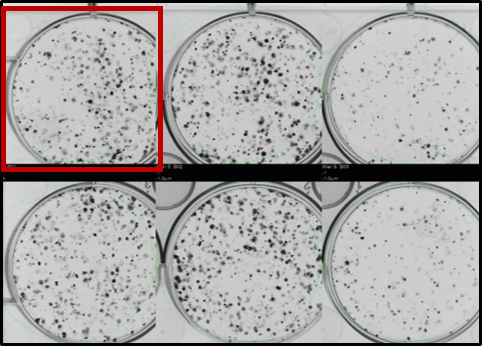

Supplement: Supplementary file 5 — Source Data Fig. 5 [file 44318_2024_43_MOESM5_ESM.zip › EMBOJ-2023-114851_SourceData_Figure5/Figure 5/5B/Images/KB2P-P2 siFen1.tif]

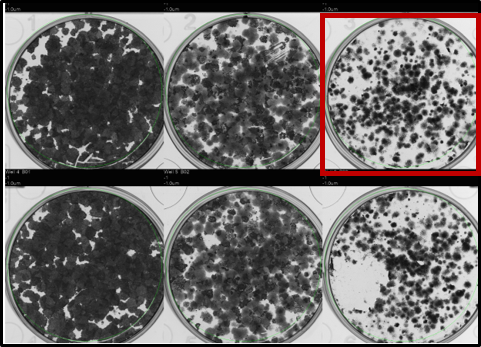

Supplement: Supplementary file 5 — Source Data Fig. 5 [file 44318_2024_43_MOESM5_ESM.zip › EMBOJ-2023-114851_SourceData_Figure5/Figure 5/5B/Images/KB2P-P2 siNT.tif]

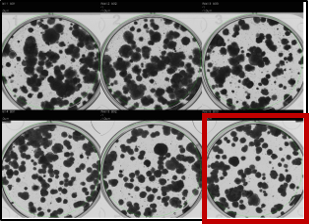

Supplement: Supplementary file 5 — Source Data Fig. 5 [file 44318_2024_43_MOESM5_ESM.zip › EMBOJ-2023-114851_SourceData_Figure5/Figure 5/5B/Images/KP-NT siFen1.tif]

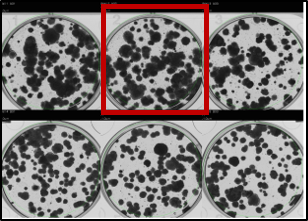

Supplement: Supplementary file 5 — Source Data Fig. 5 [file 44318_2024_43_MOESM5_ESM.zip › EMBOJ-2023-114851_SourceData_Figure5/Figure 5/5B/Images/KP-NT siNT.tif]

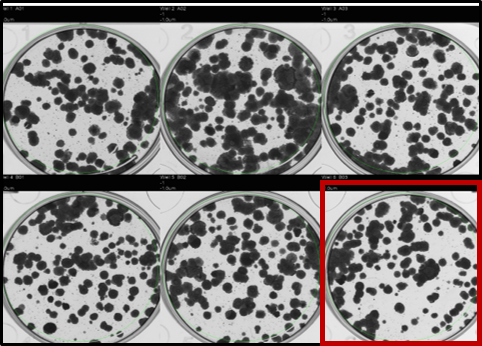

Supplement: Supplementary file 5 — Source Data Fig. 5 [file 44318_2024_43_MOESM5_ESM.zip › EMBOJ-2023-114851_SourceData_Figure5/Figure 5/5B/Images/KP-P1 siFen1.tif]

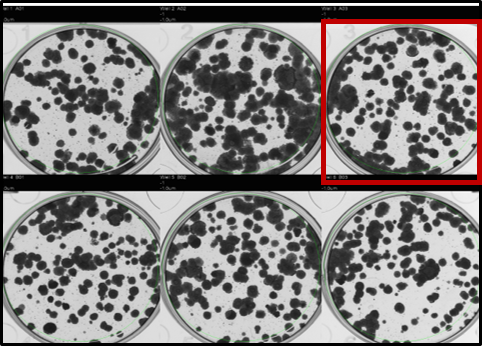

Supplement: Supplementary file 5 — Source Data Fig. 5 [file 44318_2024_43_MOESM5_ESM.zip › EMBOJ-2023-114851_SourceData_Figure5/Figure 5/5B/Images/KP-P1 siNT.tif]
