# Supplementary material for: PARG-deficient tumor cells have an increased dependence on EXO1/FEN1-mediated DNA repair
Source: EMBO J. 2024 Feb 15;43(6):6. doi: 10.1038/s44318-024-00043-2 (PMC10943112; doi:10.1038/s44318-024-00043-2)
Supplement: Supplementary file 8 — Expanded View Figures [file 44318_2024_43_MOESM8_ESM.pdf]

Expanded View Figures

Figure EV1. *Parg*$^{-/-}$ and PDDX-004-treated KB2P cells show increased PAR levels and are resistant to the PARPi olaparib.

(**A**) Sanger sequencing fragments of KB2P-NT, KB2P-P1 and KB2P-P2, corresponding to the targeted sgRNA sequences (sgRNA3-1, sgRNA3-2, sgRNA9) and the subsequent flanking regions, which confirm the successful introduction of deleterious mutations in the mouse *Parg*. (**B**) CTB-based viability quantification and representative images of the long-term clonogenic assay of the KB2P-NT, KB2P-P1 and KB2P-P2, in the absence or presence of 50 and 100 nM of olaparib. The data are representative for three independent experiments, shown as mean ± SD of replicates, two-tailed *t* test, $^{**}P < 0.01$, $^{***}P < 0.001$. (**C**) Intensity quantification and representative images of PAR immunofluorescence in KB2P-NT, KB2P-P1 and KB2P-P2 cells, 30 min following the treatment with 0.01% MMS, with or without 1.5 h pre-treatment of 500 nM olaparib. The data are representative of three independent experiments and are shown as mean ± SD of $n = 3$, two-tailed *t* test, $^{***}P < 0.001$, $^{****}P < 0.0001$. Scale bar 50 µm. (**D**) STRING analysis network of the 221 gene hits in the screen analysis. The 5 main gene clusters are indicated and the related KEGG pathways are annotated. (**E**) Table showing the pathways implicated in Cluster 1 from (**D**), which consists of the top 12 gene hits. (**F**) CTB-based viability quantification and representative images of the long-term clonogenic assay of the KB2P cells in the absence or presence of 50 and 100 nM of olaparib combined with 0, 100 and 500 nM PDDX-004. Data are representative for three independent experiments, shown as mean ± SD of replicates, two-tailed *t* test, $^{**}P < 0.01$. (**G**) Intensity quantification and representative images of PAR immunofluorescence in the KB2P cells after treatment with 0.01% MMS, with or without 1.5 h pre-treatment with 1 µM PDDX-004. Data are shown as mean ± SD of triplicates, two-tailed *t* test, $^{****}P < 0.0001$. Scale bar 100 µm.

© The Author(s)

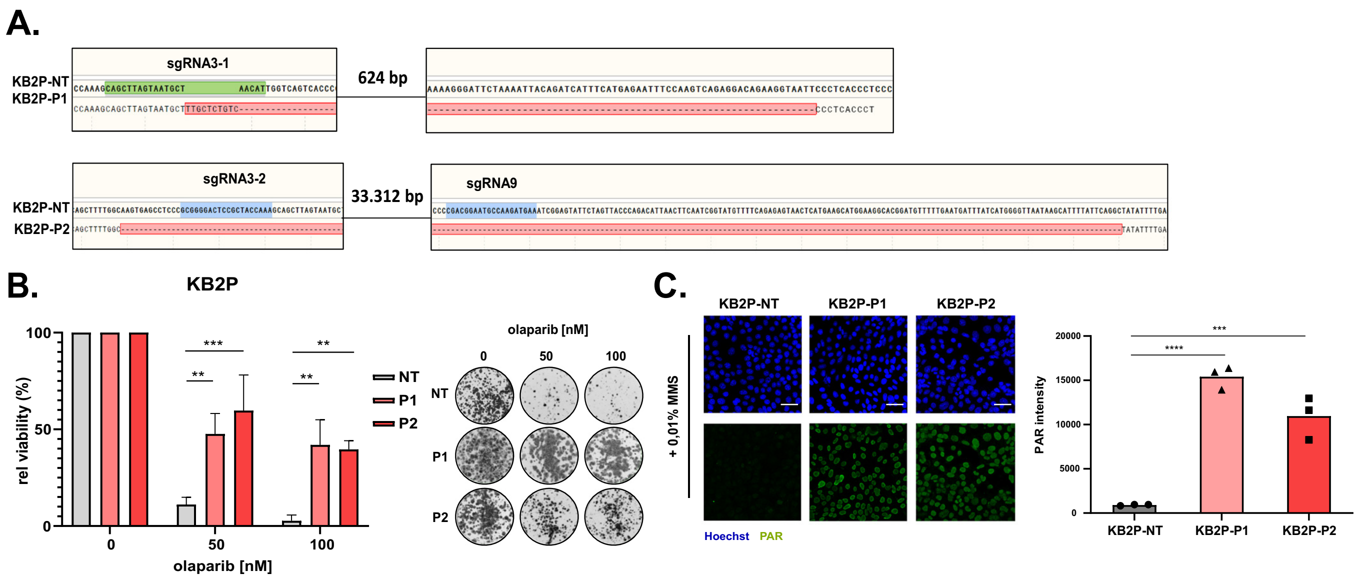

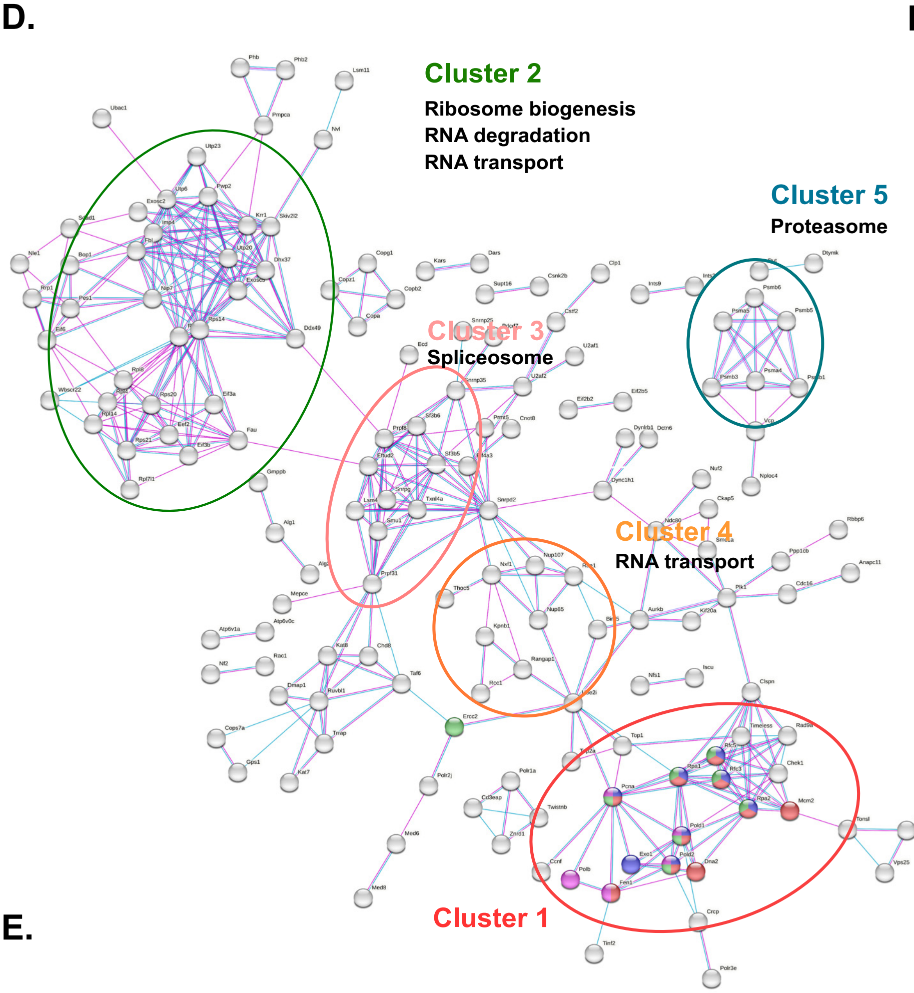

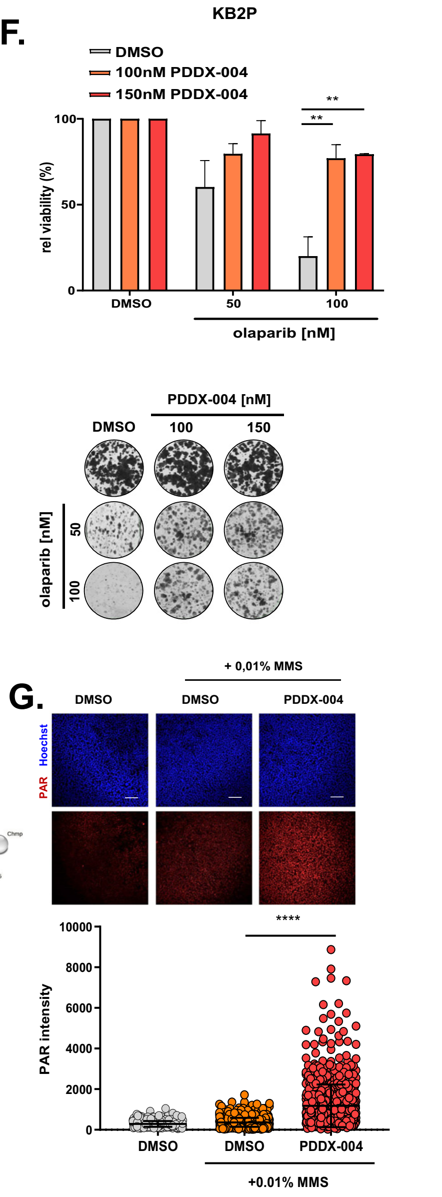

E.

KEGG pathway	Description	Count in network	FDR	
mmu03430	Mismatch repair	8 of 22	6.67e-08	
mmu03030	DNA replication	10 of 35	5.91e-09	
mmu03420	Nucleotide excision repair	8 of 43	2.43e-06	
mmu03410	Base excision repair	5 of 33	0.0012	

 © The Author(s)

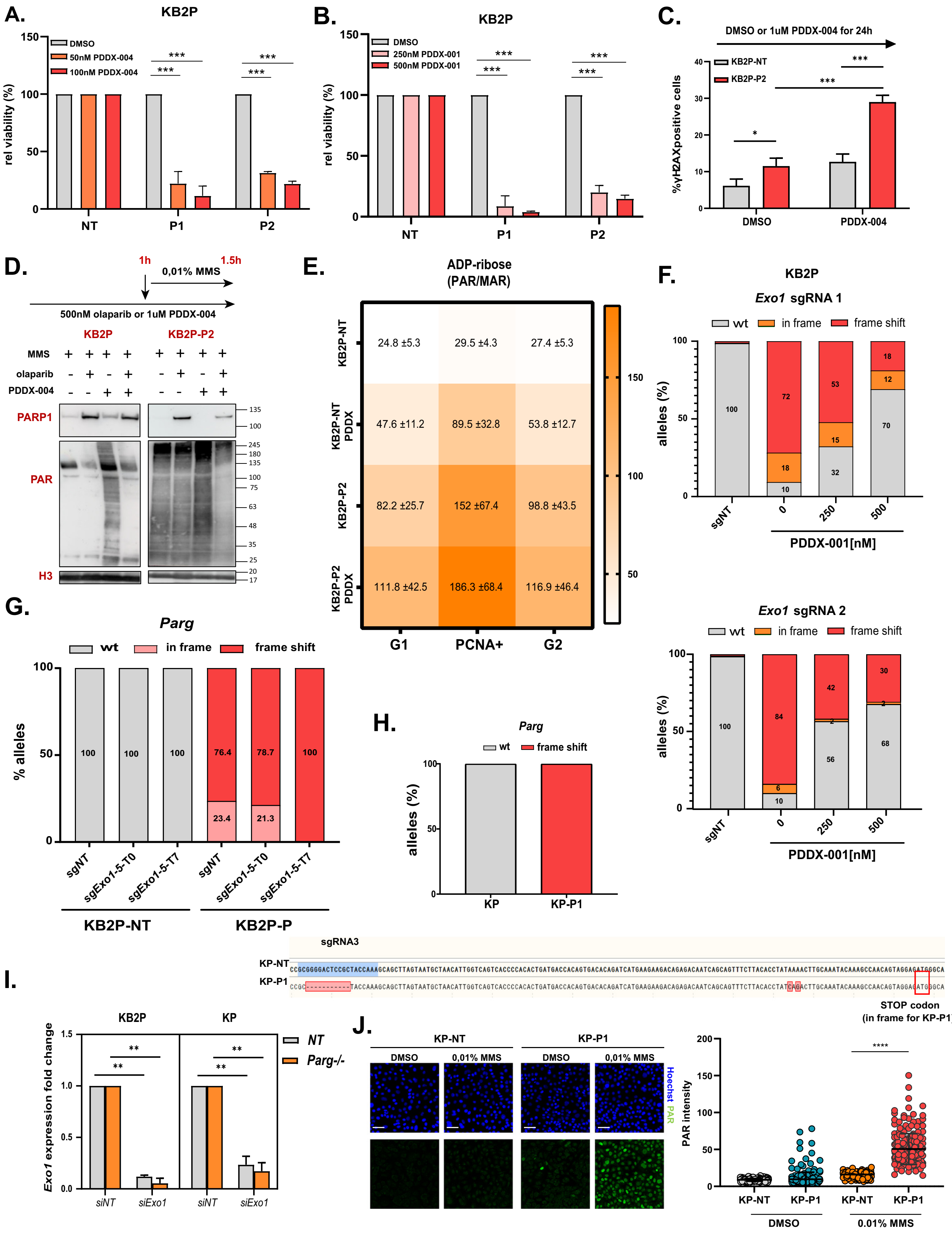
A.
KB2P
DMSO
50nM PDDX-004
100nM PDDX-004
rel viability (%)
NT
P1
P2
B.
KB2P
DMSO
250nM PDDX-001
500nM PDDX-001
rel viability (%)
NT
P1
P2
C.
DMSO or 1uM PDDX-004 for 24h
KB2P-NT
KB2P-P2
%γH2AXpositive cells
DMSO
PDDX-004
D.
1h
1.5h
0,01% MMS
500nM olaparib or 1uM PDDX-004
KB2P
KB2P-P2
MMS
olaparib
PDDX-004
PARP1
PAR
H3
E.
ADP-ribose (PAR/MAR)
KB2P-NT
KB2P-NT PDDX
KB2P-P2
KB2P-P2 PDDX
24.8 ±5.3
29.5 ±4.3
27.4 ±5.3
47.6 ±11.2
89.5 ±32.8
53.8 ±12.7
82.2 ±25.7
152 ±67.4
98.8 ±43.5
111.8 ±42.5
186.3 ±68.4
116.9 ±46.4
G1
PCNA+
G2
F.
KB2P
Exo1 sgRNA 1
wt
in frame
frame shift
alleles (%)
sgNT
PDDX-001[nM]
Exo1 sgRNA 2
G.
Parg
wt
in frame
frame shift
% alleles
sgNT
sgExo1-5-T0
sgExo1-5-T7
KB2P-NT
KB2P-P
H.
Parg
wt
frame shift
alleles (%)
KP
KP-P1
I.
sgRNA3
KP-NT
KP-P1
STOP codon
(in frame for KP-P1)
KB2P
KP
NT
Parg-/-
Exo1 expression fold change
siNT
siExo1
J.
KP-NT
KP-P1
DMSO
0,01% MMS
Hoechst
PAR
PAR intensity
DMSO
0.01% MMS

Figure EV2. PDDX inhibitor treatment and *Exo1* depletion are lethal for *Parg*$^{-/-}$;*Brca2*$^{-/-}$;*p53*$^{-/-}$ cells.

(**A**) CTB-based quantification of the long-term viability assay of the KB2P-NT, KB2P-P1 and KB2P-P2 cells, in the absence or presence of 50 and 100 nM of PDDX-004. Data are representative for three independent experiments, shown as mean ± SD of replicates, two-tailed *t* test ***$P < 0.001$. (**B**) CTB-based quantification of the long-term viability assay of the KB2P-NT, KB2P-P1 and KB2P-P2 cells, in the absence or presence of 250 and 500 nM PDDX-001. The data are representative for three independent experiments, shown as mean ± SD of replicates, two-tailed *t* test ***$P < 0.001$. (**C**) IF analysis of γH2AX foci in KB2P-NT and KB2P-P2 cells, after 24 h treatment with 1 μM of PDDX-004. Data are representative of three independent experiments shown as mean ± SD of $n = 3$, two-tailed *t* test *$P < 0.05$, ***$P < 0.001$. (**D**) Immunoblot analysis of PARP1, PAR and Histone 3 (H3)in chromatin-bound fractions of KB2P-NT and KB2P-P2 cells upon 1.5 h treatment with DMSO, 500 nM olaparib or 1 μM PDDX-004, with 0,01% MMS treatment added for the last 30 min. (**E**) Heatmap representing the ScanR quantification of median nuclear intensities of anti-ADP-ribose (PAR/MAR) immunofluorescence of KB2P-NT and KB2P-P2 cells following 30 min incubation with or without 1 μM PDDX-004. Data are shown as mean ± SD of $n = 3$. (**F**) Allelic modification rates of *Exo1* in KB2P cells upon targeting with two independent sgRNA sequences and following treatment with 0, 250 and 500 nM PDDX-001 for 1 week. (**G**) Allelic modification rates of *Parg* in KB2P-NT and KB2P-P (polyclonal *Parg*$^{-/-}$) cells upon sgRNA-mediated targeting of *Exo1*, at day 1 after puromycin selection (T0) or after 7 days in culture (T7)- corresponding to Fig. 2B. Evaluated by TIDE analysis. (**H**) Allelic modification rates of *Parg* in the sgParg3-targeted locus of KP-NT and KP-P1 (up) and Sanger sequencing fragments of KP-NT and KP-P1 containing the the targeted sgRNA3 sequence in mouse *Parg* and the formed in-frame STOP codon sequence (down). (**I**) RT-qPCR analysis of *Exo1* expression in KB2P-NT, KB2P-P2, KP-NT and KP-P1 cells. Data are shown as mean ± SD of $n = 3$, two-tailed *t* test, **$P < 0.01$. (**J**) Intensity quantification and representative images of PAR immunofluorescence in the KP-NT and KP-P1 cells 30 min after treatment with 0.01% MMS. Data are shown as mean ± SD of triplicates, two-tailed *t* test, ****$P < 0.0001$. Scale bar 50 μm.

© The Author(s)

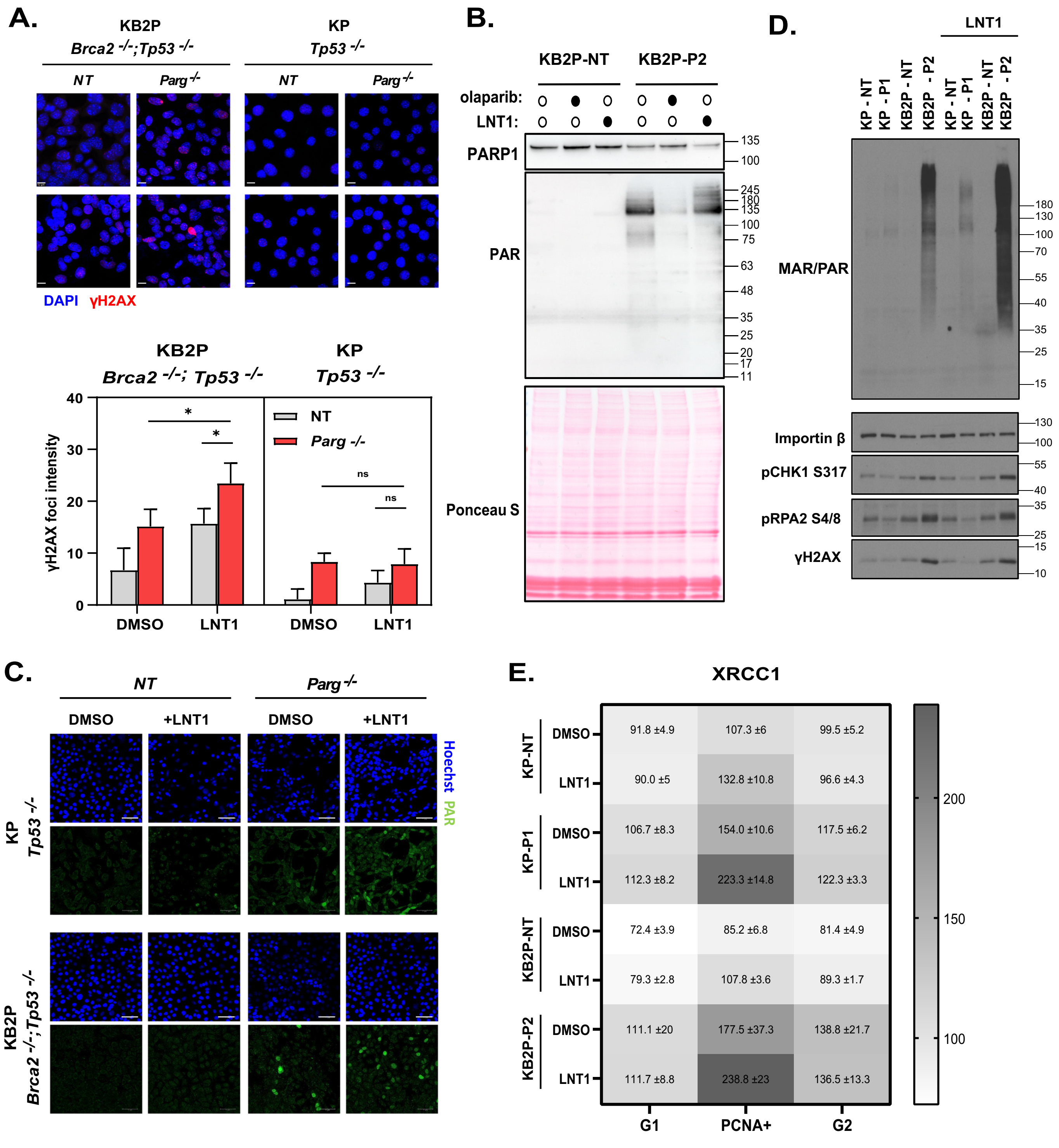

Figure EV3. LNT1 treatment results in increased DNA damage but not increased PARP1 trapping in *Parg*$^{-/-}$;*Brca2*$^{-/-}$;*p53*$^{-/-}$ cells.

(**A**) IF analysis and representative images of γH2AX foci in KB2P-NT, KB2P-P2, KP-NT and KP-P1 cells, before (upper panels) and after (lower panels) 2 h treatment with 10 μM of LNT1. Data representative of three independent experiments and are shown as mean ± SD of $n = 3$, two-tailed t test ns, non-significant, $^*P < 0.05$. Positive cells >= 10 foci. Scale bar 100 μm. (**B**) Immunoblot analysis of PARP1 and PAR in chromatin-bound fractions of KB2P-NT and KB2P-P2 cells upon 2 h treatment with DMSO, 1 μM olaparib or 10 μM LNT1. (**C**) Representative images of PAR immunofluorescence in KB2P-NT, KB2P-P2, KP-NT and KP-P1 cells, 2 h after treatment with 10 μM LNT1. Scale bar 50 μm. Related to Fig. 4B. (**D**) Immunoblot analysis MAR/PAR, pCHK1 S317, pRPA2 S4/8 and γH2aX in lysates of KB2P-NT, KB2P-P2, KP-NT and KP-P1 cells after 30 min treatment using DMSO or 10 μM LNT1. (**E**) Heatmap representing the ScanR quantification of median nuclear intensities of XRCC1 immunofluorescence of KB2P-NT, KB2P-P2, KP-NT and KP-P1 cells following 30 min incubation with 10 μM LNT1. Data are shown as mean ± SD of $n = 3$. Source data are available online for this figure.

© The Author(s)

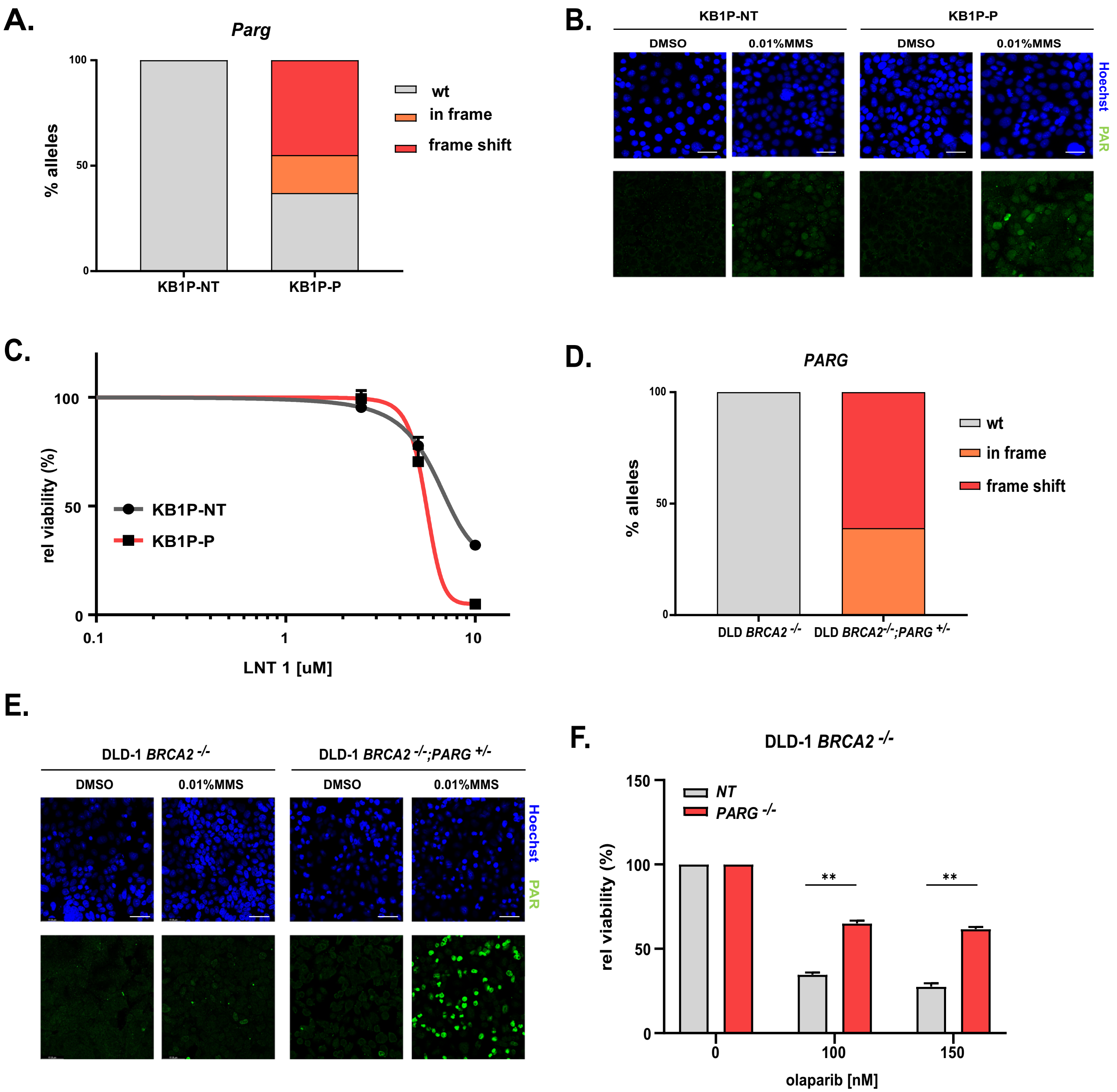

Figure EV4. PARG-deficient DLD-1 BRCA2(-/-) and KB1P cells show increased PARylation and sensitization to LNT1 treatment.

(**A**) Allelic modification rates of mouse *Parg* in the *sgParg3* locus of KB1P and KB1P cells in which *Parg* was depleted. (**B**) PAR immunofluorescence of KB1P and KB1P PARG-deficient cells following 30 min of treatment with 0.01% MMS. Scale bar 50 μm. (**C**) CTB-based viability quantification and representative images of the long-term clonogenic assay of the KB1P-NT, KB1P PARG ko cells in the presence of 0, 2.5, 5 and 10 μM of LNT1. Data are representative for three independent experiments, shown as mean ± SD of replicates. (**D**) Allelic modification rates of human *PARG* in the *sgParg7* locus of DLD-1 *BRCA2*$^{-/-}$ and DLD-1 *BRCA2*$^{-/-}$*;PARG*$^{-/-}$ cells. (**E**) PAR immunofluorescence of DLD-1 *BRCA2*$^{-/-}$ and DLD-1 *BRCA2*$^{-/-}$*;PARG*$^{-/-}$ cells following 30 min of treatment with 0.01% MMS. Scale bar 50 μm. (**F**) CTB-based viability quantification of the long-term clonogenic assay of the DLD-1 *BRCA2* $^{-/-}$ and DLD-1 *BRCA2* $^{-/-}$;PARG$^{-/-}$ cells in the presence of 0, 100 and 150 nM of olaparib. Data are shown as mean ± SD of replicates, two-tailed *t* test $^{**}P < 0.01$.

© The Author(s)
